# Urine complement-related proteins in IgA nephropathy and IgA vasculitis nephritis, possible biomarkers of disease activity

**DOI:** 10.1093/ckj/sfae395

**Published:** 2024-12-03

**Authors:** Mazdak Sanaei Nurmi, Laura Pérez-Alós, Peter Garred, Bengt Fellström, Katja Gabrysch, Sigrid Lundberg

**Affiliations:** Department of Medical Specialist Care, Nephrology Clinic, Danderyd University Hospital, Stockholm, Sweden; Department of Clinical Sciences, Karolinska Institutet, Danderyd University Hospital, Stockholm, Sweden; Laboratory of Molecular Medicine, Department of Clinical Immunology, Section, Copenhagen University Hospital, Rigshospitalet, Copenhagen, Denmark; Laboratory of Molecular Medicine, Department of Clinical Immunology, Section, Copenhagen University Hospital, Rigshospitalet, Copenhagen, Denmark; Department of Clinical Medicine, Copenhagen University Hospital Rigshospitalet, Copenhagen, Denmark; Department of Medical Sciences, Renal Medicine, Uppsala University Hospital, Uppsala, Sweden; Uppsala Clinical Research Center, Uppsala University, Uppsala, Sweden; Department of Medical Specialist Care, Nephrology Clinic, Danderyd University Hospital, Stockholm, Sweden; Department of Clinical Sciences, Karolinska Institutet, Danderyd University Hospital, Stockholm, Sweden; MedTechLabs, BioClinicum, Karolinska University Hospital, Solna, Sweden

**Keywords:** biomarkers, complement, glomerulonephritis, IgA nephropathy, vasculitis

## Abstract

**Introduction:**

The activation of the complement system plays an important role in the pathogenesis of IgA nephropathy (IgAN). Our primary aim was to evaluate a range of complement-related proteins, including pentraxin-3 (PTX-3), in blood and urine at diagnosis and their association with disease activity in the kidney biopsy, eGFR, albuminuria, and outcome. Our secondary aim was to compare the same biomarkers between patients with IgAN and IgA vasculitis with renal involvement (IgAVN).

**Methods:**

In a longitudinal Swedish cohort of 96 patients with IgAN (*n* = 65) or IgAVN (*n* = 31), with a median follow-up time of 10.8 years, we analysed mainly lectin-pathway-related proteins and PTX-3 in plasma and urine (u) samples stored at the time of kidney biopsy. Outcome was defined by the GFR slope or by the combined outcome of 50% loss of eGFR or end-stage kidney disease (ESKD).

**Results:**

Patients with detectable vs undetectable u-PTX-3 and u-mannose-binding lectin (MBL) more frequently had mesangial hypercellularity, endocapillary proliferation, and crescents in their kidney biopsy. u-C4c levels were higher in patients with advanced tubulointerstitial fibrosis, and u-C4c was also an independent predictor of a more severe eGFR slope. There were no differences in the levels of biomarkers between patients with IgAN and IgAVN.

**Conclusion:**

u-PTX-3 and u-MBL might be biomarkers of an active proliferative stage of the disease, while higher u-C4c levels indicate more chronic lesions in both IgAN and IgAVN. These results must, however, be confirmed in larger and multiethnic cohorts.

## Graphical Abstract

**Graphical Abstract:**
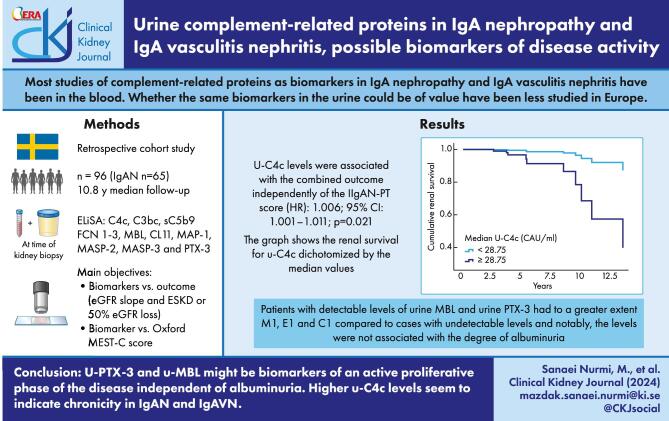

KEY LEARNING POINTS **What was known:**

- The complement system plays an important role in the pathogenesis of IgA nephropathy (IgAN) and IgA vasculitis with renal involvement (IgAVN).
- There is a need for non-invasive biomarkers to individualize follow-up and treatment of patients.
- There is a lack of studies regarding complement-related proteins in the urine as potential biomarkers in IgAN and IgAVN, as well as differences in biomarkers between the disorders.

**This study adds:**

- Patients with detectable vs not detectable u-PTX-3 and u-MBL more frequently had mesangial hypercellularity, endocapillary proliferation, and crescents in their kidney biopsy.
- Higher u-C4c levels were seen in patients with more pronounced tubulointerstitial fibrosis, and the levels were positively associated with the degree of albuminuria and disease progression.
- No differences in biomarkers and their associations were seen between patients with IgAN and IgAVN.

**Potential impact:**

- The study indicates that u-PTX-3 and u-MBL might be markers of disease activity independent of eGFR and albuminuria.
- We confirmed in a European cohort an earlier study in a Chinese population by showing that increased u-C4 split products can be markers of chronic lesions and predictive of outcome.
- The investigated complement-related proteins do not seem to be useful biomarkers for distinguishing patients with IgAN and IgAVN.

## BACKGROUND

IgA nephropathy (IgAN) is the most common primary glomerulonephritis worldwide [1]. Up to 50% of patients reach end-stage kidney disease (ESKD) within 20–30 years of diagnosis and life expectancy is reduced by 6–10 years [2–5]. Complement dysregulation, especially within the alternative and lectin pathways, is suggested to play key roles in the pathophysiology of the disease [5, 6]. Consequently, this has led to several ongoing phase 2 and 3 study trials with complement inhibitors [5]. Since the development of the international risk prediction tool for the prognosis of IgAN (IIgAN-PT) [7], it has been difficult to prove an additional impact of any single biomarker on renal outcome. As the inflammatory activity in IgAN can vary over time there is a need for non-invasive biomarkers to guide individual follow-up and treatment during the disease course.

The diagnosis of IgAN is currently based exclusively on kidney biopsy findings, which do not reflect the whole individual risk profile. Patients in Pacific Asia, compared with Europe, tend to have more progressive disease [8, 9], and a recent large genome-wide association study confirmed several ethnic differences in the IgAN risk loci, including those involved in complement regulation [10]. This highlights the importance of studying populations in different parts of the world.

Numerous studies on complement factors as potential biomarkers in IgAN have been published and recently comprehensively reviewed [11, 12]. Most of them were conducted in pacific Asia and only a few included complement analyses in urine [6, 13–16]. Pentraxin-3 (PTX-3) is a pattern recognition molecule in the same protein family as C-reactive protein (CRP) and is produced in various extrahepatic tissues and blood cells in response to inflammation and infection [17]. PTX-3 binds C1q, mannose-binding lectin (MBL), ficolin-1 (FCN-1), and -2 (FCN-2), thus activating the complement system through the classical pathway and lectin pathway. In addition, it might bind Factor H (FH) to inhibit alternative pathway activation. On the other hand, by tying to Factor H-related proteins (FHR proteins) it has also been shown to activate the alternative pathway [18]. In IgAN, mesangial cells were shown to both produce and activate PTX-3 [19]. A previous urine (u) PTX-3 study among patients with lupus nephritis found increased levels in patients with active versus inactive disease [20]. Whether this is true in IgAN has not been studied in any larger European cohort.

The primary aim of our study was to investigate, together with PTX-3, a wide range of circulating and urine complement-related proteins, mainly of the lectin pathway at the time of kidney biopsy, and to correlate the results to histopathological findings, characterized by the Oxford MEST-C classification, baseline clinical characteristics, and outcome variables. Since little is known about potential differences in the role of complement activation between patients with IgAN and IgA vasculitis nephritis (IgAVN), we also aimed to perform comparative analyses between these diseases. Our investigations are based on a Scandinavian population where, until now, only a few studies have been carried out with a focus on potential biomarkers for complement activation.

## MATERIALS AND METHODS

### Study design

In a prospectively followed Swedish IgAN and IgAVN cohort of 266 adult patients recruited between 1994 and 2014 and previously described by Ebbestad *et al*. [21], we selected 96 patients with IgAN (*n* = 65) or IgAVN (*n* = 31).

Inclusion criteria for patients with IgAN:

- Representative kidney biopsy (≥8 glomeruli) and available MEST-C.
- Unthawed plasma samples ±6 months from the day of the kidney biopsy.
- Unthawed plasma and, if available, urine samples 1–3 years after the biopsy.

Exclusion criteria for patients with IgAN and IgAVN:

- Type 1 diabetes, diabetic retinopathy, or findings of diabetic nephropathy in the kidney biopsy.
- Secondary IgAN or IgAVN.
- ESKD at kidney biopsy.
- Malignancy at diagnosis or follow-up.

In concordance with prior findings in our cohort [21], IgAN patients were grouped into three risk groups based on the 5-year risk calculated according to IIgAN-PT. High risk was defined as >12%, medium risk as 4%–12%, and low risk as <4%. To expand the number of patients, we reviewed our cohort and identified seven more patients with kidney biopsies eligible for MEST-C scoring, who were included. We aimed to prioritize high-risk patients and excluded some of the earliest cases with low risk for progression and short follow-up (*n* = 6). In total, 14 patients with low risk, 25 with medium risk, and 26 with high risk were included.

**Figure 1:**
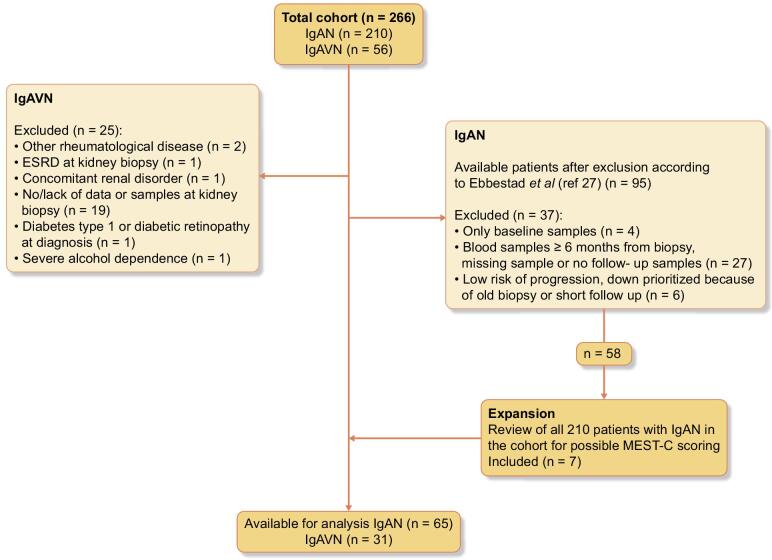
Flow diagram of study participants.

Only IgAVN patients with a kidney biopsy-verified diagnosis were included. MEST-C was not mandatory. All cases with available plasma and/or urine samples at biopsy were enrolled. The biopsies of six patients with IgAVN could not be MEST-C scored due to unrepresentative material. All biopsies in the study were classified by the same single nephropathologist. Figure 1 summarizes the patient selection.

Plasma samples and clinical data from 80 population-based healthy controls had earlier been collected from the general population in 2008 and we selected 60 of these, matched by age and gender.

All participants had signed written consent, and the study was conducted in accordance with the Declaration of Helsinki. The study was approved by the Regional, and since 2019 National, Medical Ethics Review Board in Stockholm, Sweden, with approval numbers Dnr 04-400T, Dnr 2010/213-32, Dnr 2013/1048-32, Dnr 2016/1472-32/3, and Dnr 2020-0385203.

### Quantification of biomarkers in EDTA plasma

Quantifications of C4c, C3bc, and soluble C5b9 (sC5b9), FCN-1, FCN-2, FCN-3, MBL, collectin (CL)-11, MBL/FCN/CL-associated protein-1 (MAP-1), MASP-2, MASP-3, and PTX-3 were performed using in-house-developed sandwich (S) ELISAs. Details are described in Supplementary Table S1. Briefly, Maxisorp Clear Flat-Bottom Immuno Nonsterile 384-Well Plates were coated with monoclonal antibodies and subsequently blocked with PBS + 0.05% Tween (PBS-T). The samples were diluted in dilution buffer and incubated, whereafter primary antibodies diluted in PBS-T were added. HRP-conjugated reagents were diluted in PBS-T and incubated. Plates were developed using TMB ONE as a substrate. Subsequently, 0.3 M H_2_SO_4_ was added to stop the reaction, and the optical density was measured using a Synergy HT absorbance reader (BioTek Instruments) at 450–630 nm.

### Quantification of complement in urine

Quantifications of C4c, C3bc, sC5b9, FCN-2, FCN-3, MBL, MAP-1, MASP-2, MASP-3, and PTX-3 were as for the plasma analysis, performed using S-ELISAs, but with minor modifications to adapt them for urine measurements (see Supplementary Table S2 for details).

### Interpolation of complement in plasma and urine

Estimation of complement component concentrations was performed using GraphPad Prism version 9.3.1. All biomarkers were interpolated into the respective calibrator (see Supplementary Tables S1 and S2 for details) using non-linear regression four-parameter curve fitting. Results were given either in complement arbitrary units (CAU)/mL, ng/mL, or µg/mL as appropriate.

### Clinical parameters

Plasma (p) creatinine was expressed in µmol/mL and eGFR calculated by CKD-EPI 2021. As some patients lacked urine albumin-to-creatinine ratio (u-AUCR) but had 24-h albuminuria quantified, we converted all u-AUCR values to 24-h UAE according to Fournier and Achard [22], to enable comparisons. The 5-year IIgAN-PT was estimated without race according to the original formula [7]. Outcome was defined as eGFR slope (average change of eGFR per year during the follow-up) for regression analyses and as the combined outcome of 50% eGFR loss or ESKD during the follow-up time.

MEST-C was performed to assess histopathological disease activity in the kidney biopsy. Due to the low number of patients with a score of 2 concerning tubulointerstitial fibrosis (T) and crescents (C), we scored them solely based on the presence of T or C (1 or 0, respectively). Mesangial hypercellularity (M), endocapillary proliferation (E), and segmental glomerulosclerosis (S) were scored according to standards [23].

### Statistical analysis

We utilized R version 4.2.2 and SPSS version 28.0.1.1 as software. The Mann–Whitney *U*-test was performed to compare medians. The *χ*^2^ or Fisher's exact test was applied for categorical vs categorical data and Spearman rank tests for correlation evaluation. Linear mixed effects models were applied for the estimation of eGFR slope using the R package ‘lme4’. We used multiple linear regression models to evaluate whether the levels of individual biomarkers could predict albuminuria, eGFR, or eGFR slope. Since many biomarkers, especially in the urine, had zero values, we fitted a model to handle the data. Biomarkers with >85% zeros were excluded in this analysis. If a biomarker had a value of 0, we fitted a model with discontinuity at zero, e.g. it entered the model with two terms, *I*(*x* = 0) and *I*(*x* > 0) · log_2_ *x*, where *I* was the indicator function that equals 1 if the term in brackets was true and 0 if it was false. If values of 0 were present and a discontinuity term was used, the presented *P*-value for the biomarker was for the overall effect of the marker. The models were adjusted for IgAN/IgAVN, age, gender, eGFR, and albuminuria (g/d), when appropriate. Cox proportional hazard models were conducted to identify if any biomarkers independent of the calculated IIgAN-PT risk score could predict the combined outcome. The biomarkers were analysed as both continuous and categorical variables, dichotomized by the medians. The results were expressed in hazard ratios (HRs) and 95% confidence intervals (CIs). *P*-values <0.05 were regarded as significant.

## RESULTS

A total of 96 patients (IgAN *n* = 65 and IgAVN *n* = 31) with a median age of 33.8 years were included. The median eGFR, albuminuria, and IIgAN-PT score among patients were 80.3 mL/min/1.73 m^2^, 1.1 g/d, and 7.7% respectively. Individuals with IgAN compared with IgAVN had a more severe eGFR slope during a median follow-up of 10.8 years, and to a higher degree S1 than those with IgAVN (*P* = 0.032). Cohort characteristics are displayed in Table 1.

**Table 1:** Study participant characteristics at kidney biopsy and follow-up.

	IgAN	IgAVN	*P*-value	Disease	Controls	*P*-value
At the time of kidney biopsy						
Patients (*n*)	65	31		96	60	
Age (years)	33.7 (28.4–44.9)	34.8 (26.6–54.6)	0.578	33.8 (28.3–47.1)	34.3 (29.8–45.2)	0.689
Gender (male)	48 (73.8%)	18 (58.1%)	0.119	66 (69.5%)	41 (68.3%)	0.956
Plasma creatinine (µmol/L)	104.0 (85.0–139.0)	85.0 (73.0–108.0)	***0.027***	102.0 (79.5–129.5)	77.0 (68.0–85.5)	**<0.001**
eGFR (mL/min/1.73 m^2^)	78.7 (57.1–97.1)	88.4 (65.3–114.6)	0.085	80.3 (60.5–99.7)	108.5 (98.2–116.1)	**<0.001**
Plasma albumin (g/L)	36.0 (33.0–39.0)	35.0 (30.0–37.5)	0.140	36.0 (32.0–38.0)	43.0 (41.0–45.0).	**<0.001**
Albuminuria (g/d)	1.2 (0.4–2.5)	1.1 (0.4–1.9)	0.445	1.1 (0.4–2.0)	0.0 (0.0–0.0)	**<0.001**
Systolic blood pressure (mmHg)	125.0 (120.0–141.0)	130.0 (120.0–150.0)	0.906	128.0 (120.0–141.5)	130.0 (120.0–140.0)	0.336
Diastolic blood pressure (mmHg)	85.0 (77.0–90.0)	80.0 (70.0–85.0)	***0.036***	80.0 (75.0–90.0)	80 (70.0–83.8)	0.075
Mean arterial pressure (mmHg)	98.3 (91.7–104.2)	95.7 (90.0–103.3)	0.241	96.7 (91.0–103.3)	93.3(86.7–103.3)	0.116
Body mass index (kg/m^2^)	25.3 (23.4–28.7)	26.1 (22.7–30.4)	0.751	25.5 (23.3–28.7)	24.4 (22.6–25.7)	**0.020**
IIgAN-PT score (%)^a^	9.0 (4.2–18.4)	5.2 (3.0–13.1)	0.116	7.7% (4.1–16.1)		
ACEi or ARBs at kidney biopsy	25 (38.7%)	12 (38.5%)	0.981	37 (38.5%)		
Immunosuppression at kidney biopsy	1 (1.5%)	4 (12.9%)	***0.036***			
Immunosuppression at sample	4 (6.2%)	6 (19.4%)	***0.048***			
Time between sample and kidney biopsy (d)	19 (0–71.5)	2 (0–51)	*0.213*	13.5 (0–62.5)		
Time between onset of disease and kidney biopsy (m)^b^		5.6 (0.5–80.0)				
Oxford MEST-C score						
Patients (*n*)	**65**	**25**		**90**		
M1	44 (67.7%)	14 (56.0%)	0.299	58 (64.4%)		
E1	16 (24.6%)	13 (52.0%)	*0.013*	29 (32.2%)		
S1	55 (84.6%)	16 (64.0%)	*0.032*	71 (78.9%)		
T1	25 (38.5%)	6 (24.0%)	0.196	31 (34.4%)		
C1	18 (27.7%)	12 (48.0%)	0.067	30 (33.3%)		
Follow-up						
Patients (*n*)	**65**	**31**				
Immunosuppression within 1 year of kidney biopsy (*n*/%)	15 (23.1%)	18 (58.1%)	***0.001***	33 (34.4%)		
Immunosuppression any time during follow-up (*n*/%)	22 (33.8%)	21 (67.7%)	***0.002***	43 (44.8%)		
ACEi or ARBs at any time (*n*/%)	64 (98.5%)	30 (96.8%)	*0.588*			
Time-average albuminuria (g/d)^c^	0.6 (0.3–1.1)	0.3 (0.2–1.1)	0.136	0.5 (0.2–1.1)		
eGFR slope (mL/min/1.73 m^2^ per year)	−2.0 (–3.3–−0.9)	−0.7 (−2.4–0.2)	*0.035*	−1.7 (−3.1– −0.5)		
Combined outcome^d^	22 (33.8%)	4 (12.9%)	*0.031*	26 (27.1%)		
Follow-up time (years)	11.4 (6.8–14.9)	9.4 (6.0–12)	0.099	10.8 (6.5–14.7)		

All values are presented as medians (Q1–Q3) or numbers/percentages when categorical.

Statistical significance was tested by the Mann–Whitney *U*-test. For categorical vs categorical analysis *χ*^2^ tests were performed. *P*-values <0.05 were considered significant (bolded in the table).

M1, presence of mesangial hypercellularity; E1, presence of endocapillary proliferation; S1, presence of segmental glomerulosclerosis; T1, presence of tubulointerstitial fibrosis/atrophy; C1, presence of crescent. ACEi, angiotensin-converting enzyme inhibitors; ARBs, angiotensin receptor blockers.

^a^Five-year risk of progression according to the international IgAN prediction tool; ^b^First symptoms of clinical diagnosis of IgA vasculitis. ^c^Time-average albuminuria (average albuminuria during the follow-up). ^d^eGFR loss of 50% or ESKD during the follow-up.

### Disease vs controls

As shown in Table 2, p-C4c, p-C3bc, p-sC5b9, p-FCN-1, p-FCN-2, and p-MAP-1 levels were all significantly higher in patients compared with controls. Concerning p-sC5b9, the difference was driven by patients with IgAVN rather than IgAN.

**Table 2:** Biomarkers in plasma and urine. Disease vs control and IgAN vs IgAVN.

Biomarker	IgAN	IgAVN	*P*-value	Disease	Control	*P*-value
Plasma						
Patients (*n*)	65	30		95	60	
C4c (CAU/mL)	2973 (2124–3735)	2483 (2050–3003)	0.134	2710 (2100–3655)	2241 (1854–3135)	**0.041**
C3bc (CAU/mL)	315 (247–367)	302 (240–389)	0.981	311 (246–368)	275 (231–317)	**0.028**
sC5b9 (CAU/mL)	8.3 (6.2–13.9)	11.5 (8.2–16.9)	0.075	9.4 (6.4–15.2)	7.1 (5.4–14.2)	**0.035**
FCN-1 (ng/mL)	310 (228–429)	372 (269–476)	0.137	334 (237–431)	225 (178–304)	**0.000**
FCN-2 (µg/mL)	2.8 (2.4–3.4)	3.0 (2.2–3.4)	0.923	2.8 (2.4–3.4)	2.3 (2.0–2.8)	**0.000**
FCN-3 (µg/mL)	12.2 (10.2–14.7)	13.2 (11.1–15.1)	0.282	12.7 (10.6–14.7)	12.4 (9.5–14.6)	0.218
MBL (ng/mL)	1197 (403–1938)	590 (126–1172)	**0.025**	1032 (322–1934)	785 (249–1480)	0.121
MBL no def (ng/mL)^a^	1268 (632–2048)	866 (448–1995)	0.136	1156 (465–1998)	863 (317–1537)	0.058
CL-11 (ng/mL)	326 (268–423)	334 (242–428)	0.789	326 (266–424)	366 (309–414)	0.092
MAP-1 (ng/mL)	229 (134–344)	170 (131–255)	0.118	210 (136–309)	157 (107–226)	**0.004**
MASP-2 (ng/mL)	793 (662–997)	737 (565–1040)	0.481	779 (649–998)	711 (590–905)	0.158
MASP-3 (ng/mL)	3247 (2883–3668)	3179 (2828–3829)	0.801	3231 (2837–3697)	3206 (2907–3709)	0.770
PTX-3 (ng/mL)	0.0 (0.0–0.0)	0.0 (0.0–0.42)	0.078	0.0 (0.0–0.0)	0.0 (0.0–0.0)	0.413
Urine						
Patients (*n*)	39	20				
C4c (CAU/mL)	35.0 (9.3–72.7)	21.7 (9.6–40.7)	0.301			
C3bc (CAU/mL)	3.9 (1.0–11.7)	4.1 (1.0–11.4)	0.835			

Values are presented as medians (Q1–Q3). The Mann–Whitney *U*-test was used for evaluation of statistical significance. *P*-values <0.05 were considered significant (bolded in the table).

^a^Patients with MBL deficiency were excluded (IgAN *n* = 6, IgAVN *n* = 7).

### IgAN vs IgAVN

Due to zero inflation, only u-C4c and u-C3bc were considered when comparing the levels of urine biomarkers between the diseases. As illustrated in Table 2, no significant differences in plasma or urine biomarker levels were observed between the diseases. These findings were consistent when patients with immunosuppression at sampling were excluded (Supplementary Table S3). Although p-MBL levels were lower in patients with IgAVN, the difference was not significant when excluding cases with p-MBL deficiency, defined as <100 ng/mL (IgAN *n* = 6, IgAVN *n* = 7).

### Urine biomarkers

Fifty five out of 59 patients with urine samples (IgAN *n* = 39/39, IgAVN *n* = 16/20) had a MEST-C score available.

#### Urine biomarkers vs MEST-C score

Due to the number of patients with unmeasurable levels in the urine, we compared the overall presence of the biomarker vs MEST-C. As seen in Table 3, individuals with detectable u-MBL (*n* = 16/55) and u-PTX-3 (*n* = 12/55) more frequently had M1, E1, and C1 compared with cases with undetectable levels. Patients with detectable u-sC5b9 (*n* = 16/55) to a greater extent had T1. Higher u-C4c levels were detected in the presence of M1 and T1.

**Table 3:** Biomarkers in urine vs Oxford MEST-C score.

		M			E			S			T			C		
		0	1	*P*-value	0	1	*P*-value	0	1	*P*-value	0	1	*P*-value	0	1	*P*-value
**Biomarker**	**Detected (D) vs not detected (ND)**															
Total *n*		40	15		35	20		14	31		36	19		37	18	
Non-quantifiable																
u-sC5bC9	ND	75% (30)	60% (9)	0.326	80% (28)	55% (11)	0.050	86% (12)	66% (27)	0.193	81% (29)	53% (10)	**0.030**	76% (28)	61% (11)	0.264
	D	25% (10)	40% (6)		20% (7)	45% (9)		14% (2)	34% (14)		19% (7)	47% (9)		24% (9)	39% (7)	
u-FCN-2	ND	93% (37)	87% (13)	0.606	97% (34)	80% (16)	0.053	93% (13)	90% (37)	1.000	89% (32)	95% (18)	0.649	95% (35)	83% (15)	0.317
	D	8% (3)	13% (2)		3% (1)	20% (4)		7% (1)	10% (4)		11% (4)	5% (1)		5% (2)	17% (3)	
u-FCN-3	ND	65% (26)	53% (8)	0.428	69% (24)	50% (10)	0.173	71% (10)	59% (24)	0.391	64% (23)	58% (11)	0.663	68% (25)	50% (9)	0.208
	D	35% (14)	47% (7)		31% (11)	50% (10)		29% (4)	41% (17)		36% (13)	42% (8)		32% (12)	50% (9)	
u-MBL	ND	80% (32)	47% (7)	***0. 022***	86% (30)	45% (9)	**0.001**	79% (11)	68% (28)	0.734	72% (26)	68% (13)	0.768	84% (31)	44% (8)	**0.003**
	D	20% (8)	53% (8)		14% (5)	55% (11)		21% (3)	32% (13)		28% (10)	32% (6)		16% (6)	56% (10)	
u-MASP-3	*ND*	93% (37)	87% (13)	0.606	97% (34)	80% (16)	0.053	93% (13)	90% (37)	1.000	94% (34)	84% (16)	0.327	95% (35)	83% (15)	0.317
	*D*	8% (3)	13% (2)		3% (1)	20% (4)		7% (1)	10% (4)		6% (2)	16% (3)		5% (2)	17% (3)	
u-PTX3	*ND*	88% (35)	53% (8)	**0.011**	89% (31)	60% (12)	**0.020**	86% (12)	76% (31)	0.709	83% (30)	68% (13)	0.303	89% (33)	56% (10)	**0.012**
	*D*	13% (5)	47% (7)		11% (4)	40% (8)		14% (2)	24% (10)		17% (6)	32% (6)		11% (4)	44% (8)	
Quantifiable	Detected (*n*/55)															
u-C4c (CAU/mL)	48/55	23.8 (2.7–54.4)	41.4 (28.8–73.1)	**0.037**	28.8 (2.7–53.2)	35.7 (15.8–103.7)	0.234	27.6 (18.5–46.0)	32.5 (9.3–72.7)	0.869	23.8 (9.9–42.3)	53.2 (13.1–207.7)	**0.034**	29.6 (9.3–69.6)	30.0 (13.6–70.7)	0.720
u-C3bc (CAU/mL)	50/55	3.3 (1.2–10.1)	6.4 (0.8–15.9)	0.438	3.0 (3.0–11.7)	6.5 (0.9–14.5)	0.327	1.4 (0.6–13.1)	4.2 (1.5–11.7)	0.363	2.1 (0.8–9.7)	6.2 (3.0–15.9)	0.076	3.0 (1.0–11.7)	6.3 (0.8–14.2)	0.484

Due to zero inflation of the urine biomarkers, quantitative analysis of all biomarkers was not possible. These biomarkers were instead classified as detectable or not.

The table describes the percentage (number) of patients with detectable vs not detectable biomarker in relation to each category of the MEST-C score.

Significance was tested by the *χ*^2^ test and Fishers’ test when appropriate.

Quantifiable biomarkers are described as medians (Q1–Q3) and significance was tested by the Mann–Whitney *U*-test. *P*-values <0.05 were considered significant (bolded in the table).

#### Urine biomarkers vs albuminuria and eGFR

In multiple linear regression models, higher u-C4c and u-C3bc levels were significantly associated with both a higher degree of albuminuria and a lower eGFR, even after several adjustments (Table 4). Levels of U-FCN-3 and U-sC5b9 were associated with the degree of albuminuria.

**Table 4:** Biomarkers in urine vs eGFR slope, albuminuria and eGFR.

		Model 1§						Model 2					
		eGFR slope		Albuminuria		eGFR		eGFR slope		Albuminuria		eGFR	
Biomarker	*N*	Coefficient (95% CI)	*P*-value	Coefficient (95% CI)	*P*-value	Coefficient (95% CI)	*P*-value	Coefficient (95% CI)	*P*-value	Coefficient (95% CI)	*P*-value	Coefficient (95% CI)	*P*-value
u-C3bc (CAU/mL)	=0: 7	0.37 (−1.81, 2.55)	0.703	1.66 (0.22, 3.11)	**<0.001**	−15.79 (−37.49, 5.91)	**0.004**	0.13 (−2.03, 2.29)	0.666	1.68 (0.18, 3.19)	**<0.001**	−16.40 (−37.12, 4.32)	**0.005**
	>0: 52	−0.13 (−0.57, 0.32)		0.67 (0.40, 0.95)		−7.27 (−11.36, −3.18)		−0.18 (−0.64, 0.29)		0.66 (0.36, 0.97)		−6.81 (−10.73, −2.89)	
u-C4c (CAU/mL)	=0: 8	−1.24 (−3.71, 1.24)	0.135	1.45 (−0.45, 3.34)	**0.013**	11.60 (−15.32, 38.53)	0.051	−2.53 (−5.01, −0.05)	**0.011**	1.73 (−0.21, 3.67)	**0.022**	3.10 (−23.19, 29.39)	**0.043**
	>0: 51	−0.34 (−0.67, −0.01)		0.38 (0.13, 0.64)		−2.12 (−5.71, 1.48)		−0.58 (−0.94, −0.22)		0.38 (0.12, 0.65)		−3.01 (−6.48, 0.46)	
u-FCN3 (ng/mL)	=0: 38	0.56 (−0.83, 1.96)	0.699	−0.46 (−1.51, 0.58)	**0.012**	2.62 (−12.82, 18.07)	0.441	0.77 (−0.63, 2.18)	0.506	−0.44 (−1.48, 0.61)	**0.020**	4.04 (−10.67, 18.74)	0.347
	>0: 21	−0.02 (−0.42, 0.38)		0.39 (0.10, 0.68)		−2.45 (−6.79, 1.89)		−0.07 (−0.51, 0.37)		0.37 (0.08, 0.67)		−2.46 (−6.59, 1.67)	
u-MBL (ng/mL)	=0: 42	0.07 (−1.35, 1.49)	0.992	−1.56 (−2.58, −0.53)	**0.003**	3.87 (−11.86, 19.60)	0.756	0.39 (−1.18, 1.97)	0.861	−1.53 (−2.57, −0.49)	**0.004**	6.33 (−8.85, 21.51)	0.481
	>0: 17	−0.03 (−0.77, 0.70)		−0.52 (−1.05, 0.01)		−2.40 (−10.51, 5.71)		−0.08 (−0.85, 0.69)		−0.55 (−1.10, −0.00)		−3.63 (−11.65, 4.38)	
u-PTX3 (ng/mL)	=0: 46	0.29 (−1.28, 1.86)	0.912	−0.36 (−1.59, 0.86)	0.458	10.97 (−6.05, 27.98)	0.451	0.45 (−1.11, 2.01)	0.847	−0.21 (−1.45, 1.04)	0.552	11.88 (−4.28, 28.04)	0.301
	>0: 13	0.07 (−0.66, 0.81)		−0.32 (−0.90, 0.27)		−1.03 (−9.10, 7.04)		−0.06 (−0.80, 0.68)		−0.30 (−0.90, 0.29)		−3.00 (−10.81, 4.81)	
u-sC5b9 (CAU/mL)	=0: 43	1.29 (−0.87, 3.44)	0.481	−1.10 (−2.59, 0.40)	**<0.001**	21.69 (−0.87, 44.25)	**0.081**	1.61 (−0.52, 3.74)	0.288	−0.84 (−2.40, 0.72)	**0.002**	21.97 (0.53, 43.40)	0.054
	>0: 16	0.17 (−0.38, 0.73)		0.30 (−0.09, 0.69)		1.49 (−4.45, 7.44)		0.17 (−0.52, 0.86)		0.34 (−0.06, 0.74)		1.42 (−4.25, 7.09)	

Multivariate linear regression models for biomarkers on log_2_ scale in urine vs eGFR slope (mL/min/1.73 m^2^ per year), albuminuria (g/d) (log_2_ scale) and eGFR (mL/min/1.73 m^2^).

Model 1: univariate analyses adjusted for IgAN/IgAVN type. Model 2: multivariate analyses adjusted for IgAN/IgAVN, age, gender, eGFR (CKD-EPI 2021) and albuminuria (g/d), when appropriate. If a biomarker was not detectable (0) for some patients, it entered the model with one constant term indicating the undetectable values and one term for detectable values. *P*-values were calculated for the overall effect of the biomarker in the model. *P*-values <0.05 were considered significant (bolded in the table).

**Figure 2:**
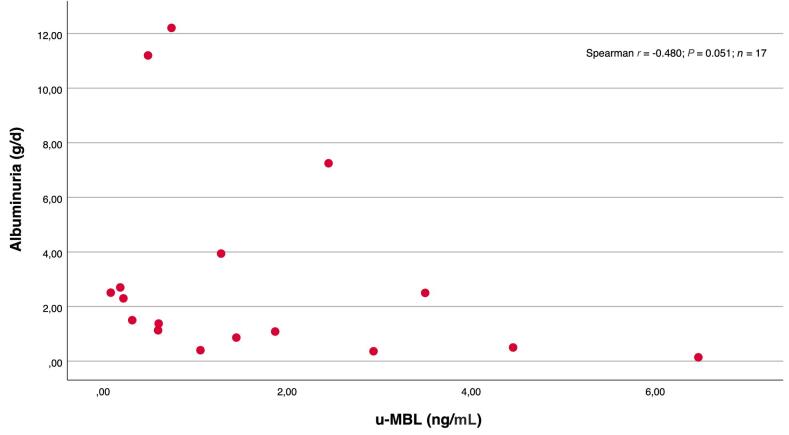
Scatterplot and correlations between u-MBL levels vs albuminuria among patients with detectable u-MBL.

**Figure 3:**
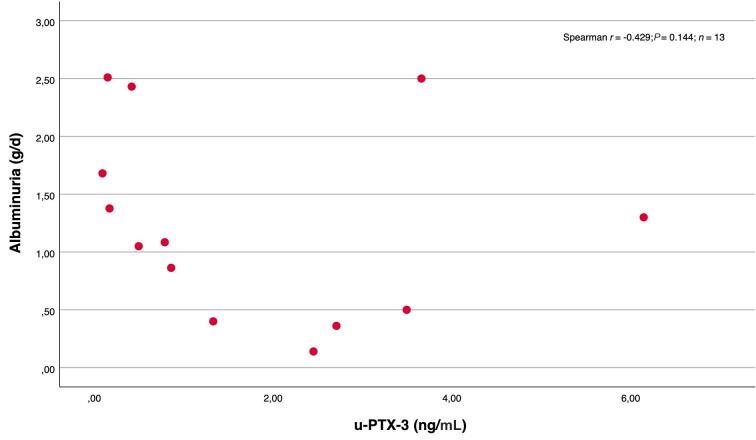
Scatterplot and correlations between u-PTX-3 levels and albuminuria in patients with detectable u-PTX-3.

Patients with detectable levels of u-MBL tended to have a higher degree of albuminuria than those with undetectable levels [1.50 (0.86–2.70) vs 0.71 g/d (0.21–1.50); *P* = 0.09]. But, as shown in Fig. 2, in patients with detectable levels of u-MBL there was rather a negative correlation between the levels of u-MBL and albuminuria, although not statistically significant (*P* = 0.051). This negative association was also seen in the multiple linear regression model, as shown in Table 4. No differences in the degree of albuminuria were seen among patients with and without u-PTX detected [1.08 (0.50–1.68) vs 0.81 g/d (0.30–1.87); *p* = 0.571]. In patients with detectable u-PTX, the levels of u-PTX were negatively correlated to the levels of albuminuria, as illustrated in Fig. 3.

Correlation plots for all biomarkers can be found in the supplement (Supplementary Figs S1–S6).

### Levels of u-C4c predicted a more severe eGFR slope and the combined outcome

**Figure 4:**
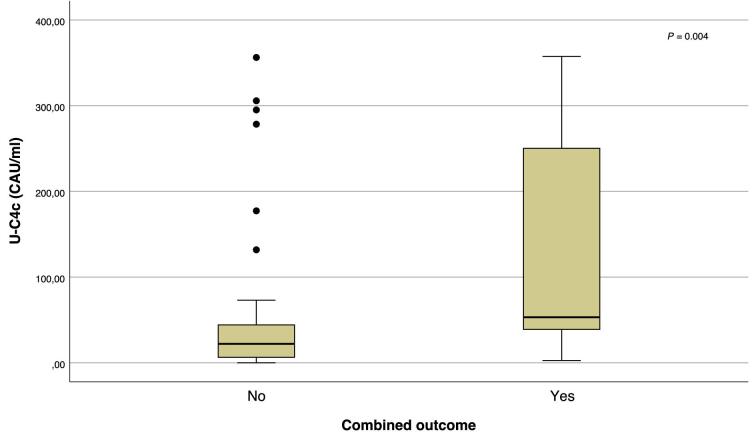
Box plots of u-C4c levels in patients with and without ESKD or 50% eGFR loss (combined outcome) during the follow up.

**Figure 5:**
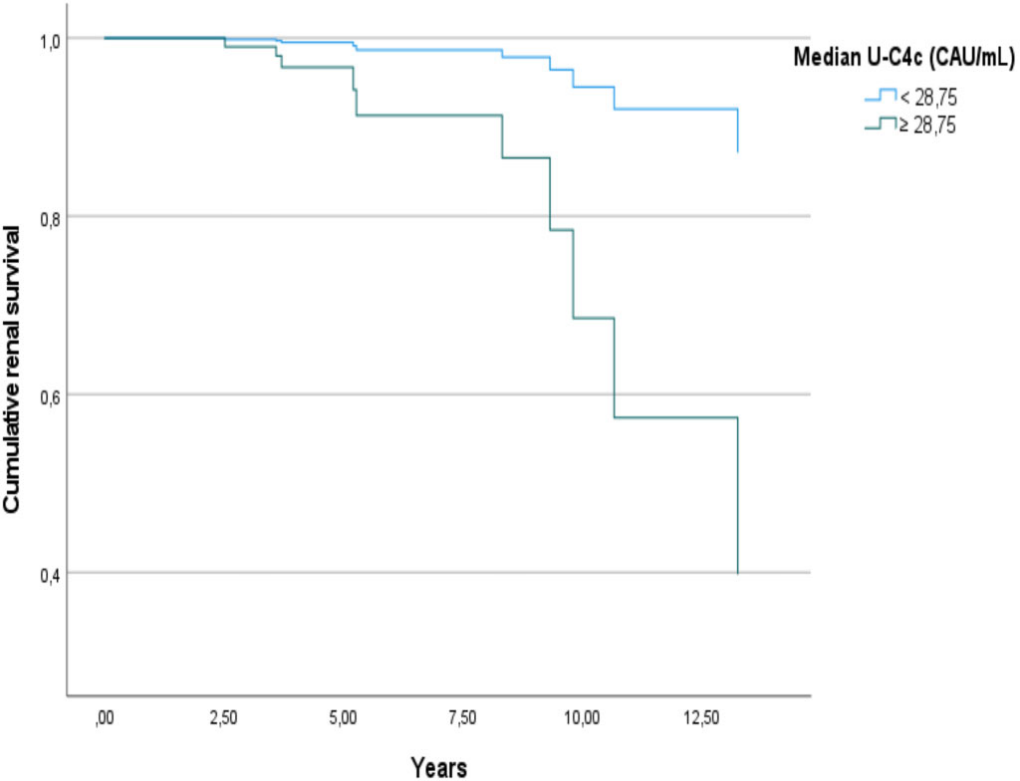
Survival plot from a Cox proportional hazard model in patients with high vs low u-C4c levels when adjusted for the calculated IIgAN-PT risk score.

In multiple linear regression models, u-C4c was the only biomarker that was independently associated with a more severe eGFR slope when adjusted for IgAN/IgAVN, age, gender, eGFR, and albuminuria (Table 4). Median u-C4c levels were, as illustrated in Fig. 4, also higher among patients that reached the combined outcome [53.2 (IQR 35.8–292.9) vs 22.1 CAU/mL (IQR 5.2–45.2); *P* = 0.004]. In a Cox proportional hazard model (Table 5), u-C4c levels were associated with the combined outcome independently of the IIgAN-PT score, but with a low hazard ratio (HR 1.006; 95% CI 1.001–1.011; *P* = 0.021). Figure 5 shows a survival plot for the combined outcome when categorizing patients into two groups, divided at the median u-C4c value of 28.75 CAU/mL, adjusted for the IIgAN-PT score.

**Table 5:** Association of urine C4c levels and progression of kidney disease.

Variable	Hazard ratio for the combined outcome (95% CI); *P*-value
	Univariate analysis
u-C4c (CAU/mL)^a^	1.002 (1.002–1.011); *P* = **0.006**
	Multivariate analysis
IIgAN-PT	1.096 (1.042–1.153); *P* < **0.001**
u-C4c (CAU/mL)	1.006 (1.001–1.011); *P* = **0.020**
	Univariate analysis
High u-C4c^b^	13.116 (1.669–103.064); *P* = **0.014**
	Multivariate analysis
High u-C4c	6.690 (0.781–57.325); *P* = 0.083
IIgAN-PT	1.077 (1.026–1.130); *P* = **0.002**

Cox proportional hazard model with and without adjustment for the calculated IIgAN-PT 5-year risk.

Combined outcome was defined as 50% eGFR loss or ESKD.

^a^Urine C4c levels as a continuous parameter.

^b^u-C4c levels dichotomized by medians to high and low group (≥28.75 vs <28.75 CAU/mL).

*P*-values <0.05 were considered significant and are bolded in the table.

### Plasma biomarkers

One sample failed to be analysed. Thus, samples from 95 patients were available (IgAN *n* = 56, IgAVN *n* = 30). In 90 patients MEST-C score had been assessed (IgA *n* = 65, IgAVN *n* = 25).

#### Biomarkers in plasma vs MEST-C score

P-MASP-3 was lower in the presence of E1 and C1. Meanwhile, p-sC5b9 and p-FCN-1 were higher in patients with T1 and C1. Details are presented in Table 6.

**Table 6:** Biomarkers in plasma vs Oxford MEST-C score (*n* = 90).

	M			E			S			T			C		
	0	1	*P*-value	0	1	*P*-value	0	1	*P*-value	0	1	*P*-value	0	1	*P*-value
C4c (CAU/mL)	2909 (2340–3680)	2201 (1784–3357)	**0.034**	3120 (2353–3809)	2158 (1630–2778)	**0.001**	3122 (1809–3400)	2643 (2115–3661)	0.881	2498 (1900–3278)	3120 (2322–4451)	0.063	2954 (2220–3735)	2347 (1855–3155)	0.050
C3bc (CAU/mL)	297 (254–364)	315 (235–394)	0.853	315 (257–378)	278 (233–344)	0.098	295 (254–364)	313 (246–367)	0.908	305 (235–364)	285 (247–370)	0.843	305 (245–366)	296 (248–364)	0.830
sC5b9 (CAU/mL)	10.5 (7.3–18.5)	8 (6–12.9)	0.141	8.3 (6.1–14.3)	12.2 (7.9–23)	**0.043**	7.3 (5.5–14.3)	9.7 (7.1–20.1)	0.183	8.3 (6–13.6)	11 (7.5–26)	**0.021**	8.2 (6.1–12.7)	13.3 (9.1–26.5)	**0.001**
FCN-1 (ng/mL)	331 (231–391)	316 (263–563)	0.257	310 (237–391)	357 (236–484)	0.394	268 (220–372)	343 (238–430)	0.083	289 (227–388)	378 (275–502)	**0.012**	289 (229–372)	395 (301–583)	**0.001**
FCN-2 (µg/mL)	2.7 (2.3–3.2)	3.0 (2.4–3.6)	0.278	2.9 (2.4–3.4)	2.6 (2.2–3.4)	0.387	2.7 (2.1–3.0)	2.8 (2.4–3.5)	0.307	2.8 (2.3–3.3)	2.8 (2.5–3.6)	0.297	2.9 (2.4–3.5)	2.6 (2.3–3.2)	0.302
FCN-3 (µg/mL)	12.7 (10.6–14.7)	11.7 (10–14.3)	0.299	12.6 (10.4–15.1)	12 (10.6–14.4)	0.870	14.5 (10.6–16.1)	12 (10.3–14.6)	0.172	12.9 (10.6–15.1)	11.8 (10.5–13.8)	0.288	12.2 (10.4–15.2)	12.6 (10.7–14.2)	0.844
MBL (ng/mL)	994 (219–1878)	1076 (448–2405)	0.252	1060 (349–1768)	1041 (351–2289)	0.536	717 (154–1706)	1102 (379–2004)	0.258	999 (219–1934)	1197 (471–2004)	0.305	1008 (305–1906)	1042 (448–2173)	0.593
MBL no def (ng/mL)	1129 (377–1934)	1127 (681–2405)	0.271	1127 (432–1775)	1197 (471–2405)	0.288	837 (219–1706)	1216 (460–2110)	0.130	1076 (349–1976)	1246 (681–2004)	0.335	1129 (426–1976)	1127 (471–2405)	0.632
CL-11 (ng/mL)	317 (268–420)	339 (268–432)	0.642	339 (268–424)	321 (267–386)	0.295	339 (268–432)	327 (268–423)	0.741	339 (270–432)	314 (257–420)	0.240	325 (265–423)	331 (282–414)	0.875
MAP-1 (ng/mL)	207 (134–282)	206 (156–370)	0.290	223 (150–309)	159 (131–282)	0.096	204 (150–259)	210 (135–315)	0.900	217 (138–293)	173 (135–309)	0.392	220 (138–284)	164 (134–336)	0.342
MASP-2 (ng/mL)	804 (664–1021)	764 (628–1007)	0.587	793 (657–995)	780 (644–1118)	0.519	764 (657–1034)	787 (649–1007)	0.992	789 (660–1022)	781 (629–1007)	0.816	796 (660–1014)	779 (649–974)	0.847
MASP-3 (ng/mL)	3228 (2820–3810)	3341 (2973–3662)	0.938	3405 (2976–3875)	2918 (2592–3408)	**0.002**	3149 (2705–3561)	3258 (2882–3776)	0.378	3305 (2838–3798)	3203 (2831–3662)	0.614	3427 (2942–3910)	3053 (2806–3366)	**0.015**

Median (Q1–Q3) levels of each biomarker in plasma are shown in relation to the presence or not of each category of the Oxford MEST-C score.

M1, presence of mesangial hypercellularity; E1, presence of endocapillary proliferation; S1, presence of segmental glomerulosclerosis; T1, presence of tubulointerstitial fibrosis/atrophy; C1, presence of crescent.

The Mann–Whitney *U*-test was performed to evaluate statistical significance. *P*-values <0.05 were considered significant (bolded in the table).

#### Plasma biomarkers vs eGFR slope, eGFR and albuminuria

As illustrated in Table 7, no plasma biomarker could predict a worse eGFR slope. However, higher p-CL-11 levels were associated with a greater degree of albuminuria and lower eGFR while p-MASP-3 and p-PTX-3 levels were associated with higher eGFR.

**Table 7:** Biomarkers in plasma vs eGFR slope, albuminuria and eGFR.

		Model 1						Model 2					
		eGFR slope		Albuminuria		eGFR		eGFR slope		Albuminuria		eGFR	
Biomarker	*N*	Coefficient (95% CI)	*P*-value	Coefficient (95% CI)	*P*-value	Coefficient (95% CI)	*P*-value	Coefficient (95% CI)	*P*-value	Coefficient (95% CI)	*P*-value	Coefficient (95% CI)	*P*-value
C3bc (CAU/mL)	>0: 95	−0.15 (−0.87, 0.57)	0.679	−0.44 (−1.00, 0.12)	0.130	0.89 (−7.44, 9.22)	0.834	−0.16 (−0.92, 0.61)	0.690	−0.46 (−1.02, 0.11)	0.115	5.20 (−2.61, 13.02)	0.195
C4c (CAU/mL)	>0: 95	0.04 (−0.62, 0.69)	0.915	−0.11 (−0.63, 0.41)	0.692	1.94 (−5.63, 9.51)	0.616	0.03 (−0.63, 0.69)	0.929	−0.05 (−0.56, 0.45)	0.831	2.45 (−4.51, 9.40)	0.493
CL-11 (ng/mL)	>0: 95	−0.28 (−1.36, 0.79)	0.609	0.17 (−0.63, 0.98)	0.671	19.84 (8.86, 30.83)	**<0.001**	−0.46 (−1.59, 0.67)	0.424	0.89 (0.05, 1.73)	**0.041**	17.60 (6.88, 28.32)	**0.002**
FCN-1 (ng/mL)	>0: 95	−0.27 (−0.97, 0.43)	0.446	0.58 (0.05, 1.12)	0.036	−5.86 (−13.80, 2.09)	0.152	−0.36 (−1.09, 0.36)	0.331	0.58 (0.05, 1.12)	0.036	−6.58 (−13.98, 0.83)	0.085
FCN-2 (µg/mL)	>0: 95	0.35 (−0.86, 1.56)	0.575	0.87 (−0.06, 1.80)	0.071	−10.84 (−24.51, 2.83)	0.124	0.46 (−0.78, 1.71)	0.467	0.58 (−0.36, 1.52)	0.229	−11.80 (−24.52, 0.91)	0.072
FCN-3 (µg/mL)	>0: 95	0.15 (−1.12, 1.42)	0.816	−0.26 (−1.27, 0.74)	0.611	−3.72 (−18.37, 10.92)	0.619	0.22 (−1.09, 1.52)	0.747	−0.43 (−1.41, 0.55)	0.391	−0.00 (−13.67, 13.67)	1.000
MAP-1 (ng/mL)	>0: 95	−0.31 (−0.90, 0.28)	0.300	−0.16 (−0.63, 0.30)	0.491	3.32 (−3.47, 10.11)	0.341	−0.33 (−0.94, 0.28)	0.294	−0.16 (−0.62, 0.30)	0.502	1.55 (−4.87, 7.97)	0.637
MASP-2 (ng/mL)	>0: 95	0.24 (−0.63, 1.10)	0.592	−0.10 (−0.79, 0.58)	0.765	−5.17 (−15.06, 4.72)	0.308	0.23 (−0.68, 1.13)	0.621	−0.21 (−0.88, 0.47)	0.550	−0.93 (−10.29, 8.44)	0.847
MASP-3 (ng/mL)	>0: 95	−0.51 (−2.22, 1.21)	0.564	−0.78 (−2.08, 0.53)	0.246	24.87 (6.43, 43.31)	**0.010**	−0.56 (−2.30, 1.18)	0.528	−0.35 (−1.66, 0.97)	0.607	19.10 (1.69, 36.50)	**0.034**
MBL (ng/mL)	=0: 7	1.65 (−1.70, 4.99)	0.587	1.54 (−1.09, 4.18)	0.510	−21.13 (−59.49, 17.23)	0.472	1.57 (−1.82, 4.96)	0.655	1.19 (−1.38, 3.76)	0.647	−24.79 (−59.99, 10.42)	0.363
	>0: 88	0.09 (−0.20, 0.38)		0.12 (−0.10, 0.35)		−2.07 (−5.38, 1.24)		0.10 (−0.19, 0.39)		0.07 (−0.15, 0.29)		−2.10 (−5.14, 0.95)	
PTX-3 (ng/mL)	=0: 76	−0.11 (−1.41, 1.18)	0.729	−1.09 (−2.06, −0.11)	0.092	17.20 (3.07, 31.33)	0.062	−0.25 (−1.59, 1.09)	0.574	−0.72 (−1.72, 0.27)	0.350	17.33 (4.35, 30.32)	**0.031**
	>0: 19	0.25 (−0.36, 0.86)		0.14 (−0.33, 0.62)		−0.15 (−7.04, 6.73)		0.33 (−0.30, 0.95)		0.10 (−0.36, 0.57)		0.96 (−5.37, 7.29)	
sC5b9 (CAU/mL)	>0: 95	0.18 (−0.36, 0.71)	0.524	0.21 (−0.20, 0.62)	0.319	−7.50 (−13.32, −1.68)	**0.013**	0.17 (−0.39, 0.74)	0.552	0.14 (−0.28, 0.57)	0.516	−5.58 (−11.29, 0.12)	0.058

Multivariate linear regression models for biomarkers on log_2_ scale in the urine vs eGFR slope (mL/min/1.73 m^2^ per year), albuminuria (g/d) (log_2_ scale) and eGFR (mL/min/1.73 m^2^).

Model 1: univariate analysis adjusted for IgAN/IgAVN type. Model 2: multivariate analysis adjusted for IgAN/IgAVN, age, gender, eGFR (CKD-EPI 2021) and albuminuria (g/d), when appropriate. If a biomarker was not detectable (0) for some patients, it entered the model with one constant term indicating the undetectable values and one term for detectable values. *P*-values were calculated for the overall effect of the biomarker in the model. *P*-values <0.05 were considered significant (bolded in the table).

## DISCUSSION

In this study we found that complement-related proteins in the urine might be potential biomarkers of disease activity in both IgAN and IgAVN. Patients with detectable levels of u-MBL and u-PTX-3 more frequently had proliferative histological changes (M1, E1, and C1) while none of these biomarkers was associated with baseline eGFR or renal outcome.

On the other hand, u-C4c levels were associated with tubulointerstitial changes, the degree of albuminuria, and more severe disease progression.

PTX-3 is a marker of endothelial activation with a potential impact on all three complement pathways. In lupus nephritis [20] higher u-PTX-3 levels were observed in patients with active disease, and in ANCA-associated vasculitis u-PTX-3 seemed to reflect disease activity better than CRP [24]. The potential of mesangial cells to activate PTX-3, alongside glomerular staining in the mesangium and endothelium in patients with IgAN, has been described earlier [19]. Although PTX-3 is non-specific for IgAN or IgAVN, our findings suggest that u-PTX-3, which might reflect local mesangial production, could be a potential biomarker for proliferative disease, independent of eGFR and albuminuria.

MBL deposition in the glomeruli has been associated with progressive IgAN, the degree of proteinuria, lower eGFR, and more severe changes in the kidney biopsy [25–27]. Furthermore, u-MBL levels were higher in all categories of MEST-C score in one study [16] and associated with crescents in another [15]. A recent biomarker study of the NEFIGAN trial found that u-MBL decreased in patients receiving active treatment [28]. MBL binding with IgA in the circulation seems to activate the complement system with subsequent MBL deposition in the kidney [29]. However, the kidneys also produce MBL locally [30, 31]. In our study the levels of u-MBL were rather negatively correlated to the degree of albuminuria, consistent with a previous Spanish study [32]. This could indicate that the appearance of MBL (and PTX-3) in urine is not proteinuria-dependent, as it might be for many other urine biomarkers, including many complement-related proteins. Particularly, the association with proliferative changes in the kidneys deserves further studies.

Wang *et al*. [33] demonstrated that u-C4d levels were associated with progressive IgAN, findings that were reproduced in a recent longitudinal study by the same group [13], in which sustained high levels predicted less response to corticosteroids and worse outcome. Similar findings were recently described in a cohort with IgAVN [34]. Our results were consistent with these findings when measuring u-C4c, though adding very little to the predictive value of the IIgAN-PT score assessed at the time of biopsy The value of u-C4c as a potential marker of irreversible kidney damage during monitoring of treatment needs to be studied further.

Current inclusion criteria for clinical trials in IgAN are the degree of proteinuria and eGFR and not specific biomarkers of inflammation. Two retrospective studies [35, 36] demonstrated that the findings of E1 and C1–2 were associated with better responsiveness to steroid therapy in IgAN, while another study found fewer responses in patients with T1–T2 compared with T0 [37]. Similar findings have been demonstrated among patients with IgAVN, in which E1 was strongly associated with initial improvement after immunosuppression but also later decline in eGFR [38]. A recent systematic review highlighted that complement activation is related to the MEST-C score [39]. The role of histological findings in decision-making in IgAN is currently being tested in a prospective trial (NCT03188887). Because kidney biopsy is considered a risky procedure [40], there is a critical need for non-invasive biomarkers reflecting the ongoing processes in the tissue.

Recently, biomarker analyses in the NEFIGAN trial [28] showed a significant drop in p-MASP-3 from baseline in patients receiving active treatment, independent of dose. We could not reproduce the findings in an earlier British study [41], which demonstrated that lower baseline p-MASP-3 was associated with progression over time. However, we found that lower baseline p-MASP-3 was associated with decreased eGFR, E1, and C1. In addition, IgAVN patients on immunosuppressive therapy at sampling had significantly lower p-MASP-3 than those without. This study did not have enough patients to draw any further conclusions on this. However, based on earlier findings [28] and the fact that corticosteroids non-specifically inhibit the complement system [42–44], one might hypothesize that underlying treatment could be one possible explanation.

Finally, our study did not find any differences in biomarker levels between patients with IgAN and IgAVN. The long latency between the onset of purpura and kidney biopsy in some IgAVN patients might have impacted these results. Many patients with IgAVN had T1 and S1 in their kidney biopsy, indicating that they probably have had their disease for a long time. We therefore cannot exclude that another cohort, with more acutely ill patients at the time of kidney biopsy, would appear different. However, among adults followed in nephrology specialist care, the clinical picture of IgAVN patients is often very similar to those with chronic IgAN, without any further extra-renal symptoms. In this clinical scenario, our study indicates that complement markers may not be likely to distinguish these two diseases. Larger patient cohorts would be needed to draw firmer conclusions.

In comparison with most previous European studies, we analysed a larger number of complement-related proteins in both plasma and urine, had a long follow-up time, and well-characterized patients. But there are also some limitations. The study cohort was relatively small and retrospective, although clinical data and samples were collected prospectively during follow-up. We could not stain for the measured complement proteins in tissue, which would have been interesting to correlate with our findings. A considerable number of patients had immunosuppression during follow-up. Hence, we cannot exclude that this might have impacted the overall outcome of the patients. Finally, the number of statistical tests performed in a study always increases the risk of spurious results.

In conclusion, our study shows that complement-related proteins in urine, particularly u-PTX-3, u-MBL, and u-C4c, might be potential biomarkers of disease activity and chronic changes in IgAN and IgAVN. These results must, however, be confirmed in more extensive and prospectively designed trials. As kidney biopsy poses risks to patients, there is a need to find non-invasive biomarkers of disease activity that could help clinicians guide follow-up and optimize treatment. Most studies have focused on biomarkers in the blood, but they might miss a potentially important local complement activation in the kidneys, which could be detected in the urine.

## Supplementary Material

sfae395_Supplemental_Files

## Data Availability

The data underlying this article are available in the article and in its online supplementary material.
