# Supplementary material for: Urine complement-related proteins in IgA nephropathy and IgA vasculitis nephritis, possible biomarkers of disease activity
Source: Clin Kidney J. 2024 Dec 3;18(1):sfae395. doi: 10.1093/ckj/sfae395 (PMC11852328; doi:10.1093/ckj/sfae395)
Supplement: sfae395_Supplemental_Files [file sfae395_Supplemental_Files.zip › 545 Supplements 241119.docx]

**Urine complement-related proteins in IgA nephropathy and IgA vasculitis nephritis, possible biomarkers of disease activity**

**Supplementary Appendix**

**Index**

**Tables**

| **Supplementary Table 1.** Description of antibodies, calibrators, and sample concentrations used for the quantification of complement components in EDTA plasma. |  |
| --- | --- |
| **Supplementary Table 2.** Description of antibodies, calibrators, and sample concentrations used for the quantification of complement components in urine.  **Supplementary Table 3**. Levels of biomarkers, IgAN vs IgAVN in patients without Immunosuppression at the time of blood sample.  **Supplementary Table 4**. Levels of albuminuria in patients with detectable vs non detectable biomarker in urine  **Supplementary Table 4.** Biomarkers in urine vs Oxford MEST-C score in patients with IgAN  **Supplementary Table 5.** Biomarkers in urine vs Oxford MEST-C score in patients with IgAVN  **Supplementary Table 6.** Cut off values for urine biomarkers |  |
| **Figures**  **Supplementary Figure 1.** Spearman correlation plot - plasma biomarkers  **Supplementary Figure 2.** Spearman correlation plot – urine biomarkers  **Supplementary Figure 3.** Scatterplot and Correlations between u-FCN-2 levels and albuminuria in patients with detectable u-FCN-2  **Supplementary Figure 4.** Scatterplot and Correlations between u-FCN-3 levels and albuminuria in patients with detectable u-FCN-3  **Supplementary Figure 5.** Scatterplot and Correlations between u-MASP-3 levels and albuminuria in patients with detectable u-MASP-3  **Supplementary Figure 6.** Scatterplot and Correlations between u-sC5b9c levels and albuminuria in patients with detectable u-sC5b9c |  |

**Supplementary Table 1.** Description of antibodies, calibrators, and sample concentrations used for the quantification of complement components in EDTA plasma.

| Assay ^a^ | Coating antibody ^b^ | | Sample dilution ^c^ | | | | | Primary antibody ^e^ | | | | | Secondary antibody ^d^ | | | | |
| --- | --- | --- | --- | --- | --- | --- | --- | --- | --- | --- | --- | --- | --- | --- | --- | --- | --- |
|  | **Clone** | **Conc (µg/ml)** | **Dilution factor** | **Calibrator used** | **Incubation temperature** | **Time (h)** | **Shaking** | **Clone** | **Conc**  **(µg/ml)** | **Incubation temperature** | **Time (h)** | **Shaking** | **Clone** | **Dilution factor** | **Incubation temperature** | **Time (h)** | **Shaking** |
| C3bc | BH6 ^e^ | 2 | 1:2000 | Zymogen Activated Serum | 4°C | 1 | No | Polyclonal Rabbit Anti-Human C3c Complement (A0062 Agilent) | 1 | Room temperature | 1:30 | Yes | Swine anti-rabbit-HRP (P0399 Agilent) | 1:2000 | Room temperature | 1 | Yes |
| C4c | 99-72-18 ^f^ | 2 | 1:300 | EDTA plasma | Room temperature | 1 | Yes | Polyclonal Rabbit Anti-Human C4c Complement (Q0369 Agilent) | 0.4 | Room temperature | 1:30 | Yes | Swine anti-rabbit-HRP (P0399 Agilent) | 1:2000 | Room temperature | 1 | Yes |
| sC5b-9 | aE11 ^g^ | 2 | 1:5 | Zymogen Activated Serum | Room temperature | 1:30 | Yes | 9C4 biotinylated ^g^ | 2 | Room temperature | 1:30 | Yes | Streptavidin-HRP (GERPN1231-2ML Sigma-Aldrich) | 1:2000 | Room temperature | 1 | Yes |
| Ficolin-1 | FCN166 ^h^ | 1 | 1:100 | EDTA plasma | 37°C | 2 | Yes | 7G1 biotinylated (HM2196-100UG Hycult Biotech) | 1 | Room temperature | 1:30 | Yes | Enhanced Streptavidin-HRP (4740N. ECO-TEK) | 1:10000 | Room temperature | 1 | Yes |
| Ficolin-2 | FCN216 ^i^ | 2 | 1:120 | EDTA plasma | Room temperature | 2 | Yes | FCN219 biotinylated ^e^ | 0.5 | Room temperature | 1:30 | Yes | Streptavidin-HRP (GERPN1231-2ML Sigma-Aldrich) | 1:2000 | Room temperature | 1 | Yes |
| Ficolin-3 | FCN334 ^j^ | 0.6 | 1:2000 | EDTA plasma | 37°C | 2 | Yes | FCN334 biotinylated ^j^ | 0.4 | Room temperature | 1:30 | Yes | Streptavidin-HRP (GERPN1231-2ML Sigma-Aldrich) | 1:2000 | Room temperature | 1 | Yes |
| MBL | HYB-131-1 ^k^ | 2 | 1:300 | EDTA plasma | Room temperature | 2 | Yes | Hyb-131-1 biotinylated ^k^ | 0.5 | Room temperature | 1:30 | Yes | Streptavidin-HRP (GERPN1231-2ML Sigma-Aldrich) | 1:2000 | Room temperature | 1 | Yes |
| PTX-3 | PTX3-66 ^l^ | 2 | 1:20 | rPTX3  spiked  EDTA plasma | Room temperature | 2 | Yes | PTX3-20 biotinylated ^l^ | 1 | Room temperature | 1:30 | Yes | Enhanced Streptavidin-HRP (4740N. ECO-TEK) | 1:25000 | Room temperature | 1 | Yes |
| MAP-1 | 20C4 ^m^ | 2 | 1:30 | EDTA plasma | Room temperature | 2 | Yes | 8B3 biotinylated ^m^ | 2 | Room temperature | 1:30 | Yes | Enhanced Streptavidin-HRP (4740N. ECO-TEK) | 1:10000 | Room temperature | 1 | Yes |
| MASP-2 | Clone 5 ^n^ | 2 | 1:120 | EDTA plasma | Room temperature | 2 | Yes | Clone 100 biotinylated ^n^ | 1.5 | Room temperature | 1:30 | Yes | Streptavidin-HRP (GERPN1231-2ML Sigma-Aldrich) | 1:2000 | Room temperature | 1 | Yes |
| MASP-3 | 7D8 ^o^ | 2 | 1:200 | EDTA plasma | Room temperature | 2 | Yes | 8B3 biotinylated ^o^ | 0.5 | Room temperature | 1:30 | Yes | Enhanced Streptavidin-HRP (4740N. ECO-TEK) | 1:10000 | Room temperature | 1 | Yes |
| Collectin-11 | CL-11-17 ^p^ | 2 | 1:100 | EDTA plasma | Room temperature | 2 | Yes | CL-11-15 biotinylated ^p^ | 2 | Room temperature | 1:30 | Yes | Streptavidin-HRP (GERPN1231-2ML Sigma-Aldrich) | 1:2000 | Room temperature | 1 | Yes |

^a^ Plates were washed three times with PBS + 0.05% Tween (8221840050, Merck) between incubation steps. Blocking with PBS + 0.05% Tween was for 1 hour at room temperature and not shaking. Plates were developed using TMB One (4380A; Kementec) as a substrate. The substrate was incubated for 15 min in the dark at room temperature and not shaking. Subsequently, 0.3 M H2SO4 was added to stop the reaction.

^b^ Coating antibodies were incubated overnight at 4°C.

^c^ Dilution buffer: PBS + 0.05% Tween + 20 mM EDTA (EDS500; Merck) + 0.5 % bovine serum + 1 µg/ml polyclonal mouse IgG (10400C; Thermo Fisher Scientific)

^d^ Dilution buffer: PBS + 0.05% Tween.

^e^ In-house produced antibody: Garred, P., T.E. Mollnes, and T. Lea, Quantification in enzyme-linked immunosorbent assay of a C3 neoepitope expressed on activated human complement factor C3. Scand J Immunol, 1988. 27(3): p. 329-35.

^f^ In-house produced antibody: Pilely, K., et al., A specific assay for quantification of human C4c by use of an anti-C4c monoclonal antibody. J Immunol Methods, 2014. 405: p. 87-96.

^g^ In-house produced antibody: Mollnes, T.E., et al., Quantification of the terminal complement complex in human plasma by an enzyme-linked immunosorbent assay based on monoclonal antibodies against a neoantigen of the complex. Scand J Immunol, 1985. 22(2): p. 197-202.

^h^ In-house produced antibody: Munthe-Fog, L., et al., Variation in FCN1 affects biosynthesis of ficolin-1 and is associated with outcome of systemic inflammation. Genes Immun, 2012. 13(7): p. 515-22.

^i^ In-house produced antibody: Hummelshoj, T., et al., Polymorphisms in the FCN2 gene determine serum variation and function of Ficolin-2. Hum Mol Genet, 2005. 14(12): p. 1651-8.

^j^ In-house produced antibody: Munthe-Fog, L., et al., Characterization of a polymorphism in the coding sequence of FCN3 resulting in a Ficolin-3 (Hakata antigen) deficiency state. Mol Immunol, 2008. 45(9): p. 2660-6.

^k^ In-house produced antibody: Garred, P., et al., Diallelic polymorphism may explain variations of the blood concentration of mannan-binding protein in Eskimos, but not in black Africans. Eur J Immunogenet, 1992. 19(6): p. 403-12.

^l^ In-house produced antibody: Bastrup-Birk, S., et al., Pentraxin-3 serum levels are associated with disease severity and mortality in patients with systemic inflammatory response syndrome. PLoS One, 2013. 8(9): p. e73119.

^m^ In-house produced antibody: Skjoedt, M.O., et al., Serum concentration and interaction properties of MBL/ficolin associated protein-1. Immunobiology, 2011. 216(5): p. 625-32.

^n^ In-house produced antibody: Götz, M.P., et al., Lectin Pathway Enzyme MASP-2 and Downstream Complement Activation in COVID-19. J Innate Immun, 2023. 15(1): p. 122-135.

^o^ In-house produced antibody: Skjoedt, M.O., et al., MBL-associated serine protease-3 circulates in high serum concentrations predominantly in complex with Ficolin-3 and regulates Ficolin-3 mediated complement activation. Immunobiology, 2010. 215(11): p. 921-31.

^p^ In-house produced antibody: Bayarri-Olmos, R., et al., Development of a Quantitative Assay for the Characterization of Human Collectin-11 (CL-11, CL-K1). Front Immunol, 2018. 9: p. 2238.

r: recombinant, h: hour, Conc: Concentration

**Supplementary Table 2.** Description of antibodies, calibrators, and sample concentrations used for the quantification of complement components in urine.

| Assay^a^ | Coating antibody ^b^ | | Sample dilution ^c^ | | | | | Primary antibody ^d^ | | | | | Secondary antibody ^d^ | | | | |
| --- | --- | --- | --- | --- | --- | --- | --- | --- | --- | --- | --- | --- | --- | --- | --- | --- | --- |
|  | **Clone** | **Conc (µg/ml)** | **Dilution factor** | **Calibrator used** | **Incubation temperature** | **Time (h)** | **Shaking** | **Clone** | **Conc**  **(µg/ml)** | **Incubation temperature** | **Time (h)** | **Shaking** | **Clone** | **Dilution factor** | **Incubation temperature** | **Time (h)** | **Shaking** |
| C3bc | BH6 ^e^ | 2 | 1:20 | Zymogen Activated Serum | 37°C | 1 | Yes | Polyclonal Rabbit Anti-Human C3c Complement (A0062 Agilent) | 1 | Room temperature | 1:30 | Yes | Swine anti-rabbit-HRP (P0399 Agilent) | 1:2000 | Room temperature | 1 | Yes |
| C4c | 99-72-18 ^f^ | 2 | 1:5 | EDTA plasma | 37°C | 1 | Yes | Polyclonal Rabbit Anti-Human C4c Complement (Q0369 Agilent) | 0.4 | Room temperature | 1:30 | Yes | Swine anti-rabbit-HRP (P0399 Agilent) | 1:2000 | Room temperature | 1 | Yes |
| sC5b-9 | aE11 ^g^ | 2 | 1:1.25 | Zymogen Activated Serum | 37°C | 2 | Yes | 9C4 biotinylated ^g^ | 2 | Room temperature | 1:30 | Yes | Streptavidin-HRP (GERPN1231-2ML Sigma-Aldrich) | 1:2000 | Room temperature | 1 | Yes |
| Ficolin-2 | FCN216 ^h^ | 2 | 1:1.25 | EDTA plasma | Room temperature | 2 | Yes | FCN219 biotinylated ^h^ | 0.5 | Room temperature | 1:30 | Yes | Streptavidin-HRP (GERPN1231-2ML Sigma-Aldrich) | 1:2000 | Room temperature | 1 | Yes |
| Ficolin-3 | FCN334 ^i^ | 0.6 | 1:4 | EDTA plasma | Room temperature | 2 | Yes | FCN334 biotinylated ^i^ | 0.4 | Room temperature | 1:30 | Yes | Streptavidin-HRP (GERPN1231-2ML Sigma-Aldrich) | 1:2000 | Room temperature | 1 | Yes |
| MBL | HYB-131-1 ^j^ | 2 | 1:2 | EDTA plasma | Room temperature | 2 | Yes | Hyb-131-1 biotinylated ^j^ | 0.5 | Room temperature | 1:30 | Yes | Streptavidin-HRP (GERPN1231-2ML Sigma-Aldrich) | 1:2000 | Room temperature | 1 | Yes |
| PTX-3 | PTX3-66 ^k^ | 2 | 1:1.25 | rPTX3  spiked  EDTA plasma | Room temperature | 2 | Yes | PTX3-20 biotinylated ^k^ | 1 | Room temperature | 1:30 | Yes | Enhanced Streptavidin-HRP (4740N. ECO-TEK) | 1:25000 | Room temperature | 1 | Yes |
| MAP-1 | 20C4 ^l^ | 2 | 1:1.25 | EDTA plasma | Room temperature | 2 | Yes | 8B3 biotinylated ^l^ | 2 | Room temperature | 1:30 | Yes | Enhanced Streptavidin-HRP (4740N. ECO-TEK) | 1:10000 | Room temperature | 1 | Yes |
| MASP-2 | Clone 5 ^m^ | 2 | 1:2 | EDTA plasma | Room temperature | 2 | Yes | Clone 100 biotinylated ^m^ | 1.5 | Room temperature | 1:30 | Yes | Streptavidin-HRP (GERPN1231-2ML Sigma-Aldrich) | 1:2000 | Room temperature | 1 | Yes |
| MASP-3 | 7D8 ^n^ | 2 | 1:1.25 | EDTA plasma | Room temperature | 2 | Yes | 8B3 biotinylated ^n^ | 0.5 | Room temperature | 1:30 | Yes | Enhanced Streptavidin-HRP (4740N. ECO-TEK) | 1:10000 | Room temperature | 1 | Yes |

^a^ Plates were washed three times with PBS + 0.05% Tween (8221840050, Merck) between incubation steps. Blocking with PBS + 0.05% Tween was for 1 hour at room temperature and not shaking. Plates were developed using TMB One (4380A; Kementec) as a substrate. The substrate was incubated for 15 min in the dark at room temperature and not shaking. Subsequently, 0.3 M H2SO4 was added to stop the reaction.

^b^ Coating antibodies were incubated overnight at 4°C.

^c^ Dilution buffer: PBS + 0.05% Tween + 20 mM EDTA (EDS500; Merck) + 0.1 % bovine serum albumin (BSA, 10735086001; Roche).

^d^ Dilution buffer: PBS + 0.05% Tween.

^e^ In-house produced antibody: Garred, P., T.E. Mollnes, and T. Lea, Quantification in enzyme-linked immunosorbent assay of a C3 neoepitope expressed on activated human complement factor C3. Scand J Immunol, 1988. 27(3): p. 329-35.

^f^ In-house produced antibody: Pilely, K., et al., A specific assay for quantification of human C4c by use of an anti-C4c monoclonal antibody. J Immunol Methods, 2014. 405: p. 87-96.

^g^ In-house produced antibody: Mollnes, T.E., et al., Quantification of the terminal complement complex in human plasma by an enzyme-linked immunosorbent assay based on monoclonal antibodies against a neoantigen of the complex. Scand J Immunol, 1985. 22(2): p. 197-202.

^h^ In-house produced antibody: Hummelshoj, T., et al., Polymorphisms in the FCN2 gene determine serum variation and function of Ficolin-2. Hum Mol Genet, 2005. 14(12): p. 1651-8.

^i^ In-house produced antibody: Munthe-Fog, L., et al., Characterization of a polymorphism in the coding sequence of FCN3 resulting in a Ficolin-3 (Hakata antigen) deficiency state. Mol Immunol, 2008. 45(9): p. 2660-6.

^j^ In-house produced antibody: Garred, P., et al., Diallelic polymorphism may explain variations of the blood concentration of mannan-binding protein in Eskimos, but not in black Africans. Eur J Immunogenet, 1992. 19(6): p. 403-12.

^k^ In-house produced antibody: Bastrup-Birk, S., et al., Pentraxin-3 serum levels are associated with disease severity and mortality in patients with systemic inflammatory response syndrome. PLoS One, 2013. 8(9): p. e73119.

^l^ In-house produced antibody: Skjoedt, M.O., et al., Serum concentration and interaction properties of MBL/ficolin associated protein-1. Immunobiology, 2011. 216(5): p. 625-32.

^m^ In-house produced antibody: Götz, M.P., et al., Lectin Pathway Enzyme MASP-2 and Downstream Complement Activation in COVID-19. J Innate Immun, 2023. 15(1): p. 122-135.

^n^ In-house produced antibody: Skjoedt, M.O., et al., MBL-associated serine protease-3 circulates in high serum concentrations predominantly in complex with Ficolin-3 and regulates Ficolin-3 mediated complement activation. Immunobiology, 2010. 215(11): p. 921-31.

r: recombinant, h: hour, Conc: Concentration

**Supplementary Table 3**. Levels of biomarkers, IgAN vs IgAVN in patients without Immunosuppression at the time of blood sample.

|  | ***IgAN*** | ***IgAVN*** | ***P-value*** |
| --- | --- | --- | --- |
| **Plasma** |  |  |  |
| **Patients (n)** | 61 | 24 |  |
| **C4c (CAU/ml)** | 3041 (2124–3735) | 2576 (2123–2930) | 0.237 |
| **C3bc (CAU/ml)** | 315 (248–367) | 313 (257–493) | 0.532 |
| **sC5b9 (CAU/ml)** | 8.3 (6.2–16.4) | 11.2 (7.6–15.0) | 0.255 |
| **FCN-1 (ng/ml)** | 310 (228–423) | 349.6 (264.5–415.5) | 0.285 |
| **FCN-2 (ng/ml)** | 2.7 (2.3–3.5) | 3.0 (2.1–3.5) | 0.770 |
| **FCN-3 (ng/ml)** | 12.2 (10.4–14.7) | 13.5 (11.3–15.4) | 0.191 |
| **MBL (ng/ml)** | 1127 (378–1906) | 685 (80–1822) | 0.215 |
| **MBL no def (ng/ml) ^a^** | 1197 (434–1955) | 866 (418–2000) | 0.347 |
| **CL-11 (ng/ml)** | 328 (269–424) | 311 (232–423) | 0.372 |
| **MAP-1 (ng/ml)** | 217 (137–337) | 184 (137–270) | 0.426 |
| **MASP-2 (ng/ml)** | 781 (660–953) | 760 (574–1083) | 0.969 |
| **MASP-3 (ng/ml)** | 3247 (2883–3668) | 3415 (2849–4071) | 0.510 |
| **Urine** |  |  |  |
| **Patients (n)** | 37 | 16 |  |
| **C4c (CAU/ml)** | 35.0 (6.0–102.3) | 22.1 (5.9–40.7) | 0.256 |
| **C3bc (CAU/ml)** | 3.9 (1.0–11.2) | 5.3 (1.2–16.6) | 0.347 |

All values presented as medians (Q1–Q3), Significance tested by Mann-Whitney U test.

^a^ patients with MBL deficiency (< 100 ng/ml) excluded (IgAN n = 57, IgAVN n = 21)

**Supplementary Table 4.** Levels of albuminuria in patients with detectable vs non detectable biomarker in urine.

| Biomarker | Detected (1) vs not (0) | Albuminuria (g/d) | P-value |
| --- | --- | --- | --- |
| u-sC5bC9 | 0 (n=43) | 0.70 (0.21 - 1.40) | 0.001 |
|  | 1 (n=16) | 2.28 (1.07 - 3.51) |  |
| u-FCN-2 | 0 (n=49) | 0.84 (0.36 - 1.70) | 0.158 |
|  | 1 (n=5) | 1.50 (1.23 - 3.94) |  |
| u-FCN-3 | 0 (n=38) | 0.90 (0.36 - 1.50) | 0.248 |
|  | 1 (n=21) | 1.00 (0.40 - 2.50) |  |
| u-MBL | 0 (n=42) | 0.71 (0.21 - 1.50) | 0.009 |
|  | 1 (n=17) | 1.50 (0.86 - 2.70) |  |
| u-MASP-3 | 0 (n=43) | 1.00 (0.40 - 1.70) | 1.000 |
|  | 1 (n=16) | 0.90 (0.36 - 2.50) |  |
| u-PTX-3 | 0 (n=43) | 0.81 (0.30 - 1.87) | 0.571 |
|  | 1 (n=16) | 1.08 (0.50 - 1.68) |  |

Albuminuria levels are presented as medians (Q1-Q3) and significance tested by the Mann-Whitney U test

**Supplementary Table 5**. Biomarkers in urine vs Oxford MEST-C score in patients with IgAN

|  |  |  | *M* | | | |  | *E* | | | |  | *S* | | | |  | *T* | | | |  | *C* | | | |  |
| --- | --- | --- | --- | --- | --- | --- | --- | --- | --- | --- | --- | --- | --- | --- | --- | --- | --- | --- | --- | --- | --- | --- | --- | --- | --- | --- | --- |
| *Score* | | | ***0*** | | ***1*** | |  | ***0*** | | ***1*** | |  | ***0*** | | ***1*** | |  | ***0*** | | ***1*** | |  | ***0*** | | ***1*** | |  |
| *Total n* | | | *31* | | *8* | |  | *29* | | *10* | |  | *7* | | *32* | |  | *24* | | *15* | |  | *29* | | *10* | |  |
| *Biomarker* | ***Nondetected (0)vs Detected (1)*** | ***Total N*** | ***N*** | ***%*** | ***N*** | ***%*** | ***P-value*** | ***N*** | ***%*** | ***N*** | ***%*** | ***P-value*** | ***N*** | ***%*** | ***N*** | ***%*** | ***P-value*** | ***N*** | ***%*** | ***N*** | ***%*** | ***P-value*** | ***N*** | ***%*** | ***N*** | ***%*** | ***P-value*** |
| u-sC5b9 | 0 | **29** | 23 | 74,2% | 6 | 75,0% | 1,000 | 23 | 79,3% | 6 | 60,0% | 0,244 | 7 | 100,0% | 22 | 68,8% | 0,158 | 20 | 83,3% | 9 | 60,0% | 0,141 | 23 | 79,3% | 6 | 60,0% | 0,244 |
|  | 1 | **10** | 8 | 25,8% | 2 | 25,0% |  | 6 | 20,7% | 4 | 40,0% |  | 0 | 0,0% | 10 | 31,3% |  | 4 | 16,7% | 6 | 40,0% |  | 6 | 20,7% | 4 | 40,0% |  |
| u-FCN-2 | 0 | **35** | 29 | 93,5% | 6 | 75,0% | 0,180 | 28 | 96,6% | 7 | 70,0% | **0,045** | 6 | 85,7% | 29 | 90,6% | 0,563 | 21 | 87,5% | 14 | 93,3% | 1,000 | 27 | 93,1% | 8 | 80,0% | 0,267 |
|  | 1 | **4** | 2 | 6,5% | 2 | 25,0% |  | 1 | 3,4% | 3 | 30,0% |  | 1 | 14,3% | 3 | 9,4% |  | 3 | 12,5% | 1 | 6,7% |  | 2 | 6,9% | 2 | 20,0% |  |
| u-FCN-3 | 0 | **23** | 20 | 64,5% | 3 | 37,5% | 0,235 | 20 | 69,0% | 3 | 30,0% | 0,060 | 6 | 85,7% | 17 | 53,1% | 0,206 | 15 | 62,5% | 8 | 53,3% | 0,740 | 20 | 69,0% | 3 | 30,0% | 0,060 |
|  | 1 | **16** | 11 | 35,5% | 5 | 62,5% |  | 9 | 31,0% | 7 | 70,0% |  | 1 | 14,3% | 15 | 46,9% |  | 9 | 37,5% | 7 | 46,7% |  | 9 | 31,0% | 7 | 70,0% |  |
| u-MBL | 0 | **29** | 26 | 83,9% | 3 | 37,5% | **0,016** | 25 | 86,2% | 4 | 40,0% | **0,009** | 6 | 85,7% | 23 | 71,9% | 0,653 | 18 | 75,0% | 11 | 73,3% | 1,000 | 25 | 86,2% | 4 | 40,0% | **0,009** |
|  | 1 | **10** | 5 | 16,1% | 5 | 62,5% |  | 4 | 13,8% | 6 | 60,0% |  | 1 | 14,3% | 9 | 28,1% |  | 6 | 25,0% | 4 | 26,7% |  | 4 | 13,8% | 6 | 60,0% |  |
| u-MASP-3 | 0 | **35** | 29 | 93,5% | 6 | 75,0% | 0,180 | 28 | 96,6% | 7 | 70,0% | **0,045** | 7 | 100,0% | 28 | 87,5% | 1,000 | 23 | 95,8% | 12 | 80,0% | 0,279 | 27 | 93,1% | 8 | 80,0% | 0,267 |
|  | 1 | **4** | 2 | 6,5% | 2 | 25,0% |  | 1 | 3,4% | 3 | 30,0% |  | 0 | 0,0% | 4 | 12,5% |  | 1 | 4,2% | 3 | 20,0% |  | 2 | 6,9% | 2 | 20,0% |  |
| u-PTX-3 | 0 | **30** | 27 | 87,1% | 3 | 37,5% | **0,009** | 25 | 86,2% | 5 | 50,0% | **0,032** | 7 | 100,0% | 23 | 71,9% | 0,169 | 21 | 87,5% | 9 | 60,0% | 0,063 | 25 | 86,2% | 5 | 50,0% | **0,032** |
|  | 1 | **9** | 4 | 12,9% | 5 | 62,5% |  | 4 | 13,8% | 5 | 50,0% |  | 0 | 0,0% | 9 | 28,1% |  | 3 | 12,5% | 6 | 40,0% |  | 4 | 13,8% | 5 | 50,0% |  |

The table describes the number and percentage of patients with detectable vs not detectable biomarker in relation to each category of the MEST-C score. Significance was tested by Chi Squared test or Fishers’ test when appropriate. P-values <0.05 were considered significant (bolded in the table).

**Supplementary Table 6**. Biomarkers in urine vs Oxford MEST-C score in patients with IgAVN

|  |  | *M* | | | |  | *E* | | | |  | *S* | | | |  | *T* | | | |  | *C* | | | |  |
| --- | --- | --- | --- | --- | --- | --- | --- | --- | --- | --- | --- | --- | --- | --- | --- | --- | --- | --- | --- | --- | --- | --- | --- | --- | --- | --- |
| *Score* | | ***0*** | | ***1*** | |  | ***0*** | | ***1*** | |  | ***0*** | | ***1*** | |  | ***0*** | | ***1*** | |  | ***0*** | | ***1*** | |  |
| Total N | | 9 | | 7 | |  | 6 | | 10 | |  | 7 | | 9 | |  | 12 | | 4 | |  | 8 | | 8 | |  |
| *Biomarker* | ***Nondetected (0)vs Detected (1)*** | ***N*** | ***%*** | ***N*** | ***%*** | ***P-value*** | ***N*** | ***%*** | ***N*** | ***%*** | ***P-value*** | ***N*** | ***%*** | ***N*** | ***%*** | ***P-value*** | ***N*** | ***%*** | ***N*** | ***%*** | ***P-value*** | ***N*** | ***%*** | ***N*** | ***%*** | ***P-value*** |
| u-sC5b9 | 0 | 7 | 77,8% | 3 | 42,9% | 0,302 | 5 | 83,3% | 5 | 50,0% | 0,307 | 5 | 71,4% | 5 | 55,6% | 0,633 | 9 | 75,0% | 1 | 25,0% | 0,118 | 5 | 62,5% | 5 | 62,5% | 1,000 |
|  | 1 | 2 | 22,2% | 4 | 57,1% |  | 1 | 16,7% | 5 | 50,0% |  | 2 | 28,6% | 4 | 44,4% |  | 3 | 25,0% | 3 | 75,0% |  | 3 | 37,5% | 3 | 37,5% |  |
| u-FCN-2 | 0 | 8 | 88,9% | 7 | 100,0% | 1,000 | 6 | 100,0% | 9 | 90,0% | 1,000 | 7 | 100,0% | 8 | 88,9% | 1,000 | 11 | 91,7% | 4 | 100,0% | 1,000 | 8 | 100,0% | 7 | 87,5% | 1,000 |
|  | 1 | 1 | 11,1% | 0 | 0,0% |  | 0 | 0,0% | 1 | 10,0% |  | 0 | 0,0% | 1 | 11,1% |  | 1 | 8,3% | 0 | 0,0% |  | 0 | 0,0% | 1 | 12,5% |  |
| u-FCN-3 | 0 | 6 | 66,7% | 5 | 71,4% | 1,000 | 4 | 66,7% | 7 | 70,0% | 1,000 | 4 | 57,1% | 7 | 77,8% | 0,596 | 8 | 66,7% | 3 | 75,0% | 0,755 | 5 | 62,5% | 6 | 75,0% | 1,000 |
|  | 1 | 3 | 33,3% | 2 | 28,6% |  | 2 | 33,3% | 3 | 30,0% |  | 3 | 42,9% | 2 | 22,2% |  | 4 | 33,3% | 1 | 25,0% |  | 3 | 37,5% | 2 | 25,0% |  |
| u-MBL | 0 | 6 | 66,7% | 4 | 57,1% | 1,000 | 5 | 83,3% | 5 | 50,0% | 0,307 | 5 | 71,4% | 5 | 55,6% | 0,633 | 8 | 66,7% | 2 | 50,0% | 0,604 | 6 | 75,0% | 4 | 50,0% | 0,608 |
|  | 1 | 3 | 33,3% | 3 | 42,9% |  | 1 | 16,7% | 5 | 50,0% |  | 2 | 28,6% | 4 | 44,4% |  | 4 | 33,3% | 2 | 50,0% |  | 2 | 25,0% | 4 | 50,0% |  |
| u-MASP-3 | 0 | 8 | 88,9% | 7 | 100,0% | 1,000 | 6 | 100,0% | 9 | 90,0% | 1,000 | 6 | 85,7% | 9 | 100,0% | 0,438 | 11 | 91,7% | 4 | 100,0% | 1,000 | 8 | 100,0% | 7 | 87,5% | 1,000 |
|  | 1 | 1 | 11,1% | 0 | 0,0% |  | 0 | 0,0% | 1 | 10,0% |  | 1 | 14,3% | 0 | 0,0% |  | 1 | 8,3% | 0 | 0,0% |  | 0 | 0,0% | 1 | 12,5% |  |
| u-PTX-3 | 0 | 8 | 88,9% | 5 | 71,4% | 0,550 | 6 | 100,0% | 7 | 70,0% | 0,250 | 5 | 71,4% | 8 | 88,9% | 0,550 | 9 | 75,0% | 4 | 100,0% | 0,529 | 8 | 100,0% | 5 | 62,5% | 0,200 |
|  | 1 | 1 | 11,1% | 2 | 28,6% |  | 0 | 0,0% | 3 | 30,0% |  | 2 | 28,6% | 1 | 11,1% |  | 3 | 25,0% | 0 | 0,0% |  | 0 | 0,0% | 3 | 37,5% |  |

The table describes the number and percentage of patients with detectable vs not detectable biomarker in relation to each category of the MEST-C score. Significance was tested by Chi Squared test or Fishers’ test when appropriate. P-values <0.05 were considered significant (bolded in the table).

| **Biomarker** | **Lower limit of detection** |
| --- | --- |
| u-C4c (CAU/ml) | 0.632 |
| u-C3bc (CAU/ml) | 0.040 |
| u-sC5b9 (CAU/ml) | 0.0139 |
| u-FCN2 (ng/ml) | 1.488 |
| u-FCN-3 (ng/ml) | 0.004 |
| u-MBL (ng/ml) | 0.074 |
| u-MASP-3 (ng/ml) | 1.154 |
| u-PTX-3 (ng/ml) | 0.014 |

**Supplementary Table 6.** Cut off values for urine biomarkers
