# Supplementary figures and images for: Urine complement-related proteins in IgA nephropathy and IgA vasculitis nephritis, possible biomarkers of disease activity
Source: Clin Kidney J. 2024 Dec 3;18(1):sfae395. doi: 10.1093/ckj/sfae395 (PMC11852328; doi:10.1093/ckj/sfae395)

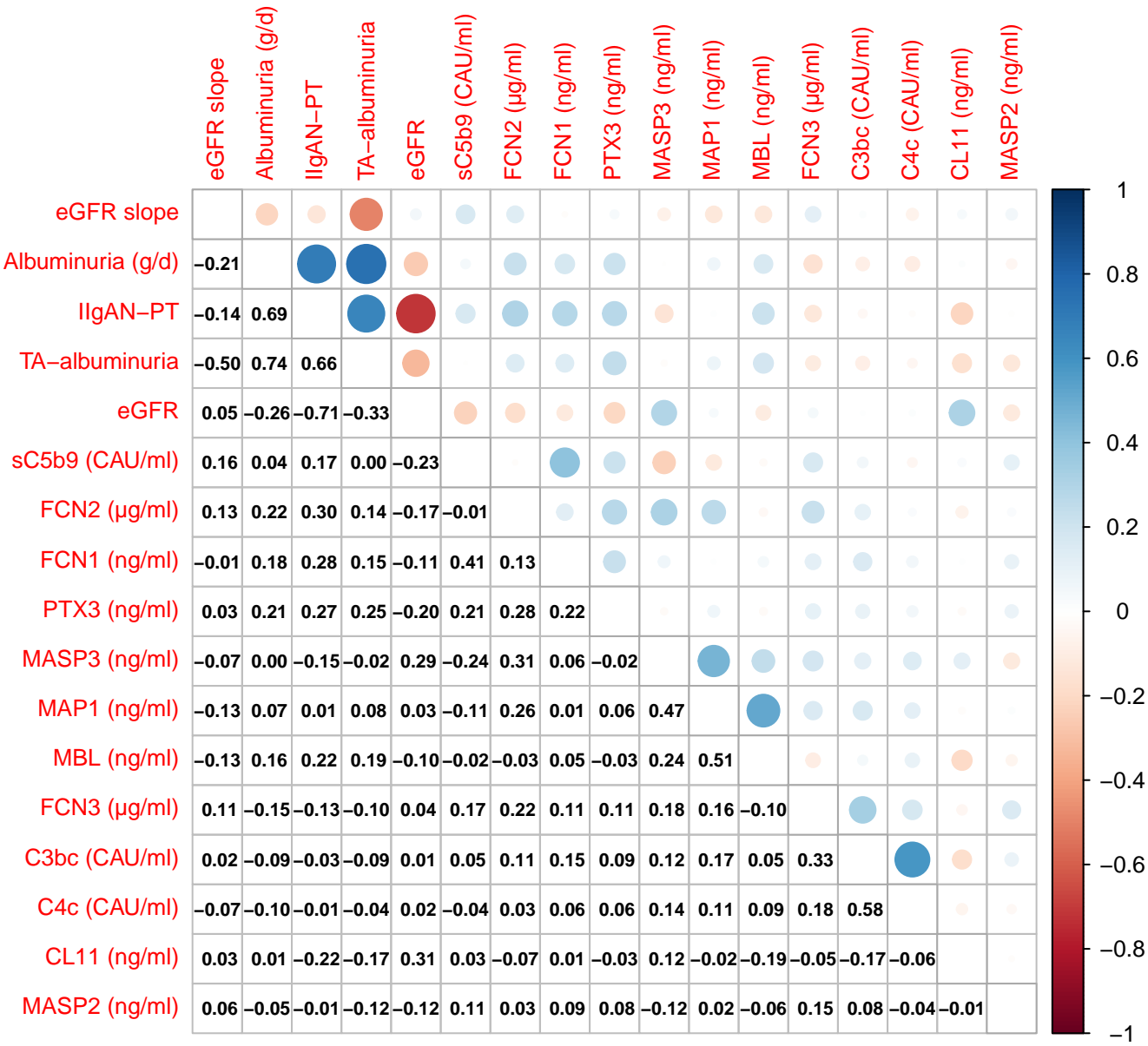

Supplement: sfae395_Supplemental_Files [file sfae395_Supplemental_Files.zip › 545 Supplement Figure 1. Spearman correlation plot - plasma.pdf]

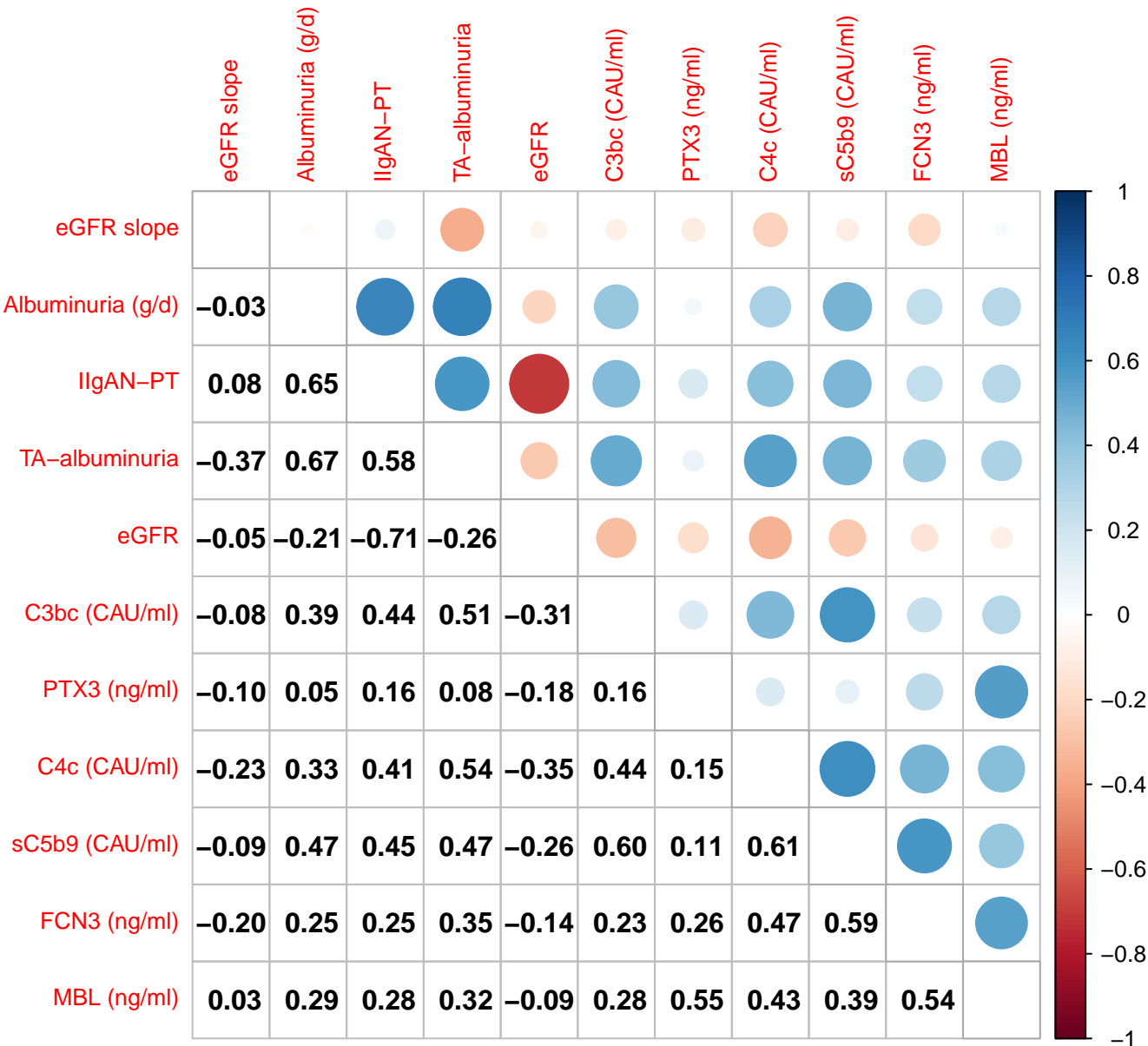

Supplement: sfae395_Supplemental_Files [file sfae395_Supplemental_Files.zip › 545 Supplement Figure 2. Spearman correlation plot - urine.pdf]

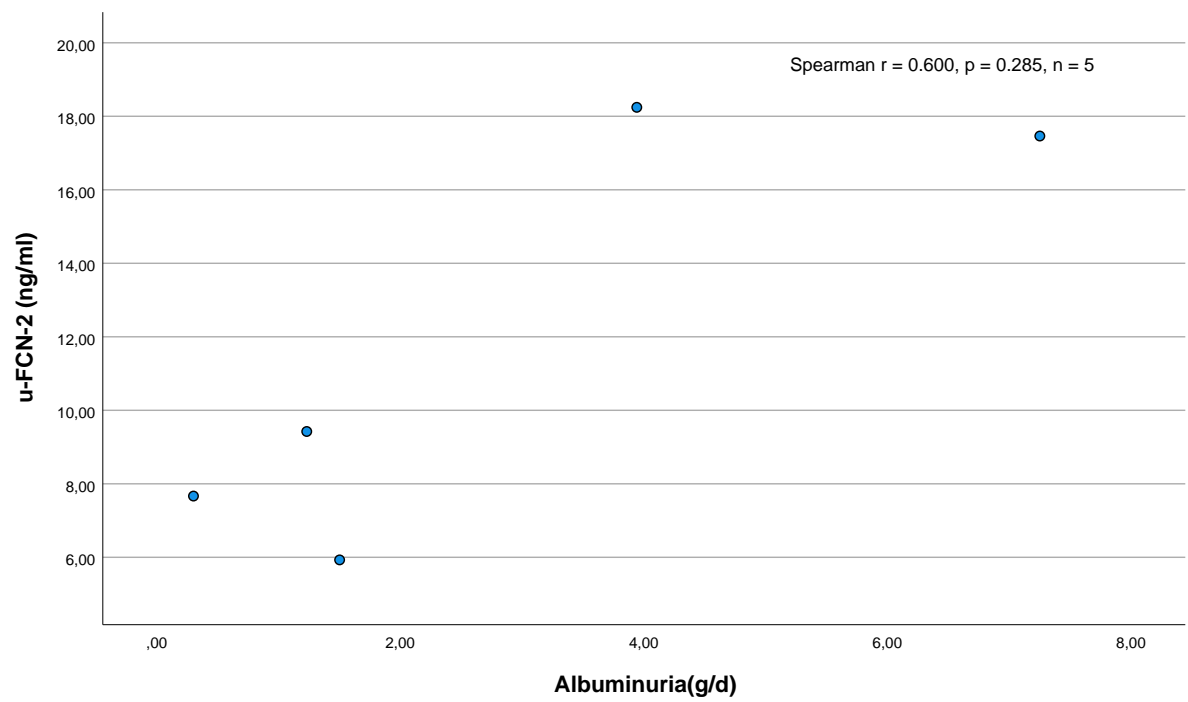

Supplement: sfae395_Supplemental_Files [file sfae395_Supplemental_Files.zip › 545 Supplement Figure 3. Scatterplot and correlation tests - Urine FCN-2 and albuminuria.pdf]

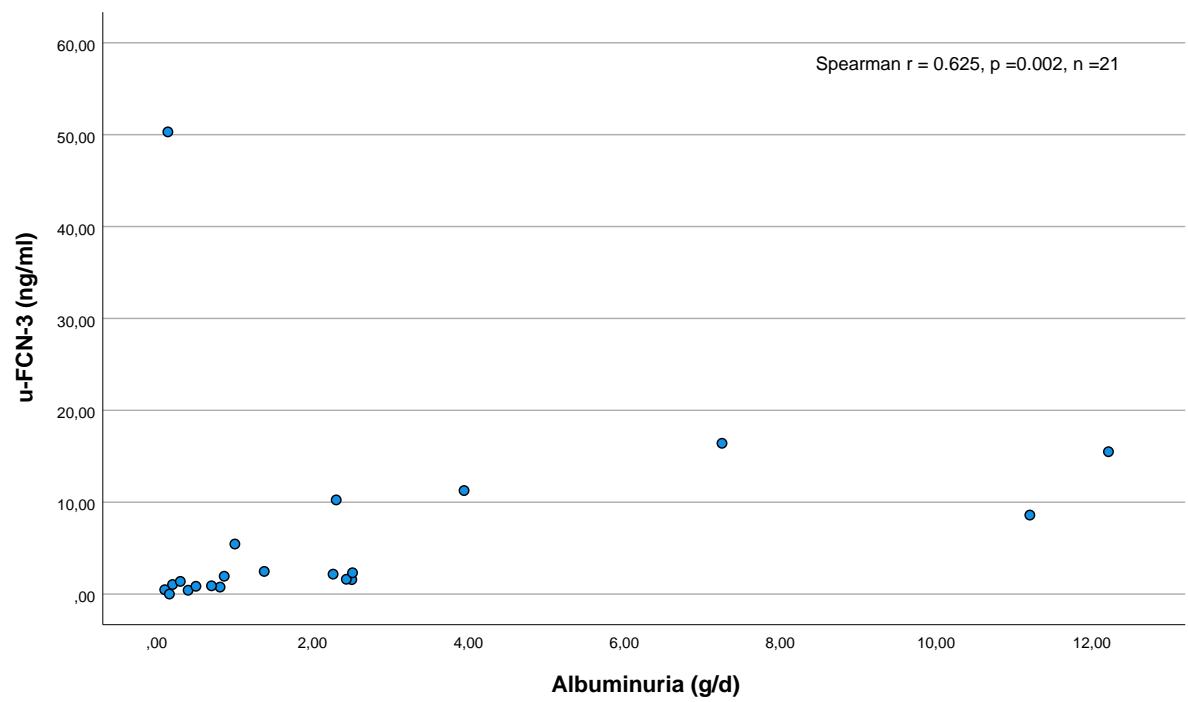

Supplement: sfae395_Supplemental_Files [file sfae395_Supplemental_Files.zip › 545 Supplement Figure 4. Scatterplot and correlation tests - Urine FCN-3 and albuminuria.pdf]

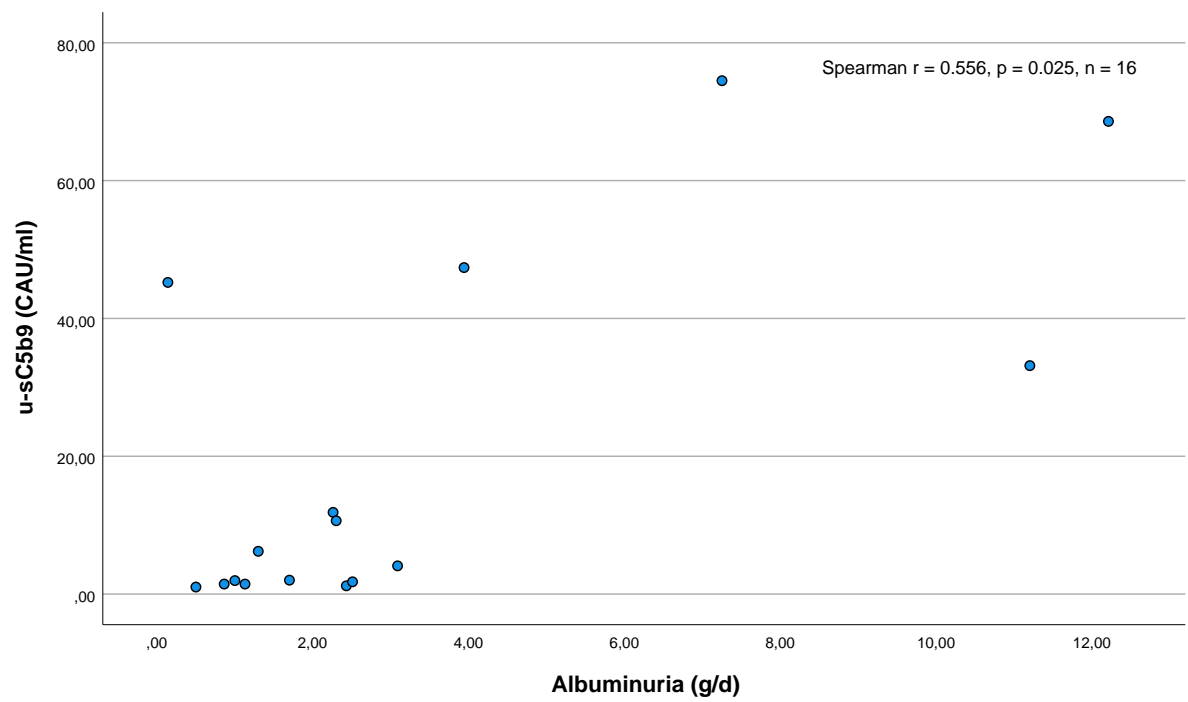

Supplement: sfae395_Supplemental_Files [file sfae395_Supplemental_Files.zip › 545 Supplement Figure 5. Scatterplot and correlation tests - Urine sC5b9 and albuminuria.pdf]

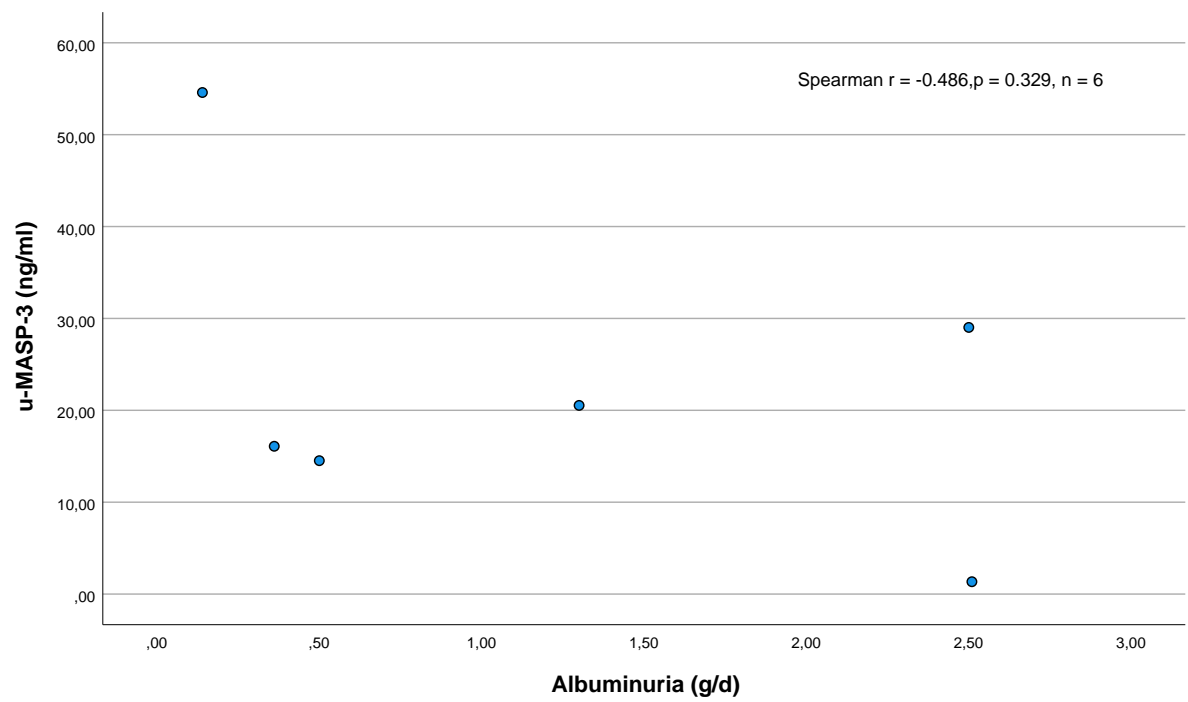

Supplement: sfae395_Supplemental_Files [file sfae395_Supplemental_Files.zip › 545 Supplement Figure 6. Scatterplot and correlation tests - Urine MASP3- and albuminuria.pdf]
